# Machine learning based prediction of diesel engine emissions and performance using hemp biodiesel enriched with nano additives

**DOI:** 10.1038/s41598-026-49243-2

**Published:** 2026-06-01

**Authors:** Anchupogu Praveen, Krupakaran Radhakrishnan Lawrence, R. Satya Meher, Dhinesh Balasubramanian, Utku Kale, Artūras Kilikevičius

**Affiliations:** 1 Department of Mechanical Engineering, Bapatla Engineering College, Bapatla, Andhra Pradesh India; 2 Department of Mechanical Engineering, Mohan Babu University, Tirupathi, Andhra Pradesh India; 3 Department of Mechanical Engineering, QIS College of Engineering & Technology, Ongole, Andhra Pradesh India; 4 Department of Port Engineering, Lithuanian Maritime Academy (LMA), Vilnius Gediminas Technical University, Klaipėda, Lithuania; 5 Department of Aeronautics and Naval Architecture, Faculty of Transportation Engineering and Vehicle Engineering, Budapest University of Technology and Economics, Műegyetem rkp. 3, Budapest, H-1111 Hungary; 6 Vilnius Gediminas Technical University, Vilnius, Lithuania

**Keywords:** Hemp biodiesel, Performance, Emission, Prediction, Decision tree, ANN, Energy science and technology, Engineering, Environmental sciences

## Abstract

The present study was focused on the experimental and machine learning approach to evaluate the diesel engine parameters behavior with hemp biodiesel blends enriched with nano additives (Al_2_O_3_, TiO_2_ and MWCNT’s) at different loads. The Hemp biodiesel was characterized by using FTIR and GC-MS analysis. The nano additives were examined through a SEM and XRD analysis for identifying the morphological structure and crystalline phases. The experimental results revealed that HMBD20 + MWCN100 fuel shows higher BTE (5.60%) and lower BSFC (18.58%) compared to HMBD20 fuel. The CO and HC pollutants for HMBD20 + TINP100 fuel were diminished by 22.24% and 16.12% but NOx emissions were enhanced by 13.7% than HMBD20 fuel at peak load. For predictive modelling, Decision Tree (DT), Support Vector Machine (SVM) and Artificial Neural Network (ANN) were employed using the engine load and fuel type as input features. The DT model exhibits a higher prediction accuracy of R^2^ 0.9899 & 0.998 and lowest RMSE of 1.085 & 0.0264 for BTE and BSFC data. The greater accuracy of R^2^ of 0.9865, 0.9899 and 0.9975 were achieved by DT model for CO, HC and Smoke emissions but NOx emissions were predicted by 0.9997 by using ANN model. The optimal condition was recorded for HMBD20 + 100 ppm MWCNT fuel at 75% load, achieving BTE (32.56%), BSFC (0.30 kg/kWhr), CO (0.12%), HC (42 ppm), NOx (980 ppm), Smoke opacity (35.2 HSU) and CO_2_ (7.48%) which confirms the strong potential of nano additive hemp biodiesel for efficient and cleaner operation of a diesel engine.

## Introduction

The demand for petroleum-based fuels was escalated drastically due to the rapid industrialization and population across the world. Among these, diesel fuel plays a key role and primary energy source for transportation, agriculture and industry purposes^1^. Recently, the rapid depletion of fossil fuels causes growing dependency on fossil fuels and severe concerns for long-term sustainability. The combustion of petroleum-based fuels contributes to environmental pollution such as particulate matter and harmful pollutants which adversely impacts the air quality and human health^2^. Biodiesel derived from non-edible feedstocks, edible sources, alcohol additives and incorporation of nanotechnology proved as a promising strategy to enhance the engine performance and diminish harmful emissions^3^. The cleaner and more efficient energy solutions with nano technology results in improved combustion and better fuel formulation. Asokan et al.^4^ prepared Hemp biodiesel using methanol and KOH to evaluate the engine performance and emission parameters. The B20 blended fuels exhibits the best performance with lesser BSFC (0.30 kg/kWhr) than diesel (0.32 kg/kWhr). In addition, pollutants like CO, HC and NOx were lowered for all hemp seed oil biodiesel blends. They concluded that B20 hemp biodiesel is a promising and efficient alternative fuel for diesel engines.

Nachippan et al.^5^ evaluates the performance of CI engine fueled with hemp biodiesel enriched with MWCNT’s. The experimental results indicate that there is a notable improvement in BTE and NOx emissions along with a decline of CO, HC and smoke emissions. They concluded that with the usage of MWCNT’s to enhance performance and stability of hemp of biodiesel as a potential result towards cleaner and efficient deployment of biodiesel. Seyyed Hassan Hosseini et al.^6^ studied the effects of CNT additives (30–90 ppm) in biodiesel blends (B5 and B10) on a CI engine. The test results at full load across 1800–2800 rpm show an improvement of power (3.67%), BTE (8.12%) and EGT (5.57%) along with diminished SFC, Soot, CO and UHC pollutants. Prabu et al.^7^ reported that JBD30A30C fuel shows higher BTE of 31% and reduction of NO by 13%, CO by 60%, UBHC by 33% and smoke emissions by 32% than biodiesel. They concluded that alumina and ceria nano particles were effective fuel borne catalysts for promising pathway towards the cleaner combustion and improved efficiency in diesel engines for the development of sustainable technology. Ghanati et al.^8^ explored the effects of TiO_2_ and SiO_2_ NPs as additives in diesel and biodiesel fuels. They reported that nano particles as additives enhanced the fuel properties like viscosity, density and cetane number including the improvement in combustion efficiency also. Additionally, NP additive fuels increase thermal efficiency and power output but reduces the fuel consumption, NOx, CO and HC emissions of diesel engines. Kolakoti^9^ investigated the diesel engine characteristics using waste cooking and neem oil biodiesel enriched with 50 and 100 ppm Al_2_O_3_ nano particles. The engine test results revealed that B10 with 50 ppm of Al_2_O_3_ NPs was achieved 32.84% of BTE and radiation loss of 32.05% at 75% load. They confirmed that nano additive (Al_2_O_3_) biodiesel blends rise combustion rate and reduction of NOx, CO, UHC and smoke emissions. Devan et al.^10^ studied a novel ternary fuel blend comprises of castor oil methyl ester, pyrolytic oil and 30 ppm graphene quantum dots (GQDs) to explore low viscosity and lubricity in pyro diesel fuels. They reported NOx and smoke emissions were increased for PCD10 fuel along with a better performance of an engine. Finally, GQD has exhibited a large surface area, biocompatibility and solubility which leads to a reduction of emissions.

Soudagar et al.^11^ reported environmental impact and effectiveness of CI engines, motivating towards the searching of an alternative fuel. They are described the key areas such as biodiesel as an alternative fuel, nano fluids application and AI/ML based optimization techniques for the biodiesel production, improving fuel characteristics and engine performance. They concluded that environmental sustainability and energy efficiency of IC engines is achieved by alternative fuels, nano technology and AI/ML integrated methods. They outlined the current limitations, identified the future research outlines for optimizing performance, minimizing emissions and suitability for real time applications. Aneesh Vijay Kale et al.^12^ conducted experiments on HCCI engines and results were noted as lowered NOx and smoke emissions with an improved efficiency. The influence of fuel characteristics such as molecular weight, H/O, C/O ratios, energy content and research octane number on the impact of HCCI combustion were studied. A total of 147 experimental data points were designed to predict combustion parameters, ISEC and pollutants. The Multi objective Pareto optimization with TOPSIS was selected for optimal blended ratio. The best optimum model was developed with a reduction of ISEC by 18% and NOx emissions by 76%. They concluded that AI-driven fuel optimization strategy is viable in enhancing the HCCI engine performance and emission control. Prabhakar Sharma et al.^13^ investigates the DF diesel engine performance using algal biodiesel along with biogas as a primary fuel. They developed ANN model using experimental values at various loads, CR’s, injection pressures and timings. The model exhibited high predictive accuracy showing R^2^ (0.9453–0.9761) for combustion and emission responses. RSM model shows an optimal condition of 84% load, 244 bar, 17.5 CR and 28 ^0^BTDC and all experimental values were validated by showing less than 9% deviation from predicted values. They concluded that ANN-RSM hybrid technique was highly effective for modelling and optimizing the diesel engine performance. Wong et al.^14^ identifies the optimal biodiesel ratio to ensure less emissions, good fuel economy and versatile engine operating conditions. By using the trained data and experimental data they modelled three ML models such as Extreme Learning Machine, Least squares-SVM and RBF Neural Network. They adopted two optimization techniques such as simulated annealing (SA) and particle swarm optimization (PSO) to propose an objective function for finding an optimal biodiesel ratio. They concluded that ELM with LT (logarithmic transformation) is superior to RBFNN and LS-SVM, additionally PSO outperforms SA in terms of SD and fit along with a satisfactory computational time.

Vijay Kumar et al.^15^ evaluates the influence of DPA (diphenylamine) antioxidant and CeO_2_ NP’s to the Jatropha (B30) biodiesel on the engine parameters. Experimental tests were designed using DoE technique to systematically assess the engine behavior. Additionally, ML techniques such MLP, RFR, KNN were adopted to predict the performance and emissions of an engine. They observed B30 + DPA50+ CeO_2_50 blend achieved 6.35% of reduction in BSFC, 8.68% decrease in NOx emissions compared to B30 fuel. Further, an improvement of BTE by 2.54% and 5.74% decrement of peak cylinder pressure also. The results demonstrate the potential of DPA and CeO_2_ in optimizing biodiesel performance and emission control. Erlin Tian et al.^16^ applied six ML algorithms (GB, XGBoost, RF, Bagging, MLNN, and DT) to predict emissions from a H_2_ gas enriched diesel engine. The models were evaluated using MAE, R^2^, MSE and AARD % with gradient boosting, XGBoost and their results were outperformed. Increased NOx by 26%, reduction of CO and CO_2_ emissions by 15–19% was observed with the addition of hydrogen (10 LPM) at peak load and 2200 rpm. XGBoost model exhibited superior predictive performance especially for CO_2_ emissions with R^2^ of 0.997 and for RF model R^2^ of 0.964. Additionally, CO and NOx emissions were also predicted by using R^2^ values for all the models and better results were obtained. Naveen et al.^17^ approached 29 ML regression and classification algorithms to predict emissions from a DF engine operating with H_2_ gas and diesel. They are considered five input parameters such as load, hydrogen concentration, diesel intake, speed and equivalence ratio to estimate the NOx, CO_2_, HC and smoke emissions. The pace regression model was achieved with a greater accuracy for CO_2_ (R^2^ of 0.9985) along with MLP regressor, RBF regressor and an alternating model tree for NOx, HC and smoke shows best correlation of R^2^ was 0.9950, 0.8958 and 0.9256, respectively. Among all, MLP regressor performs consistently with high accuracy of R^2^ value which is above 0.85 for all emissions. The results confirmed that ML algorithms can provide a reliable prediction for emission control in dual-fuel engines.

Aqueel Ahmad et al.^18^ adopted BBD (Box-Behnken Design) method with L46 orthogonal array to optimize the DF engine parameters using five inputs such as load, CR, blend ratio, nano particle concentration and % of HHO. Additionally, six ML techniques (DTR, ABR, ETR, GBR, LGBM and XGBR) were used to predict engine performance and emissions. They reported that RSM based regression equation identifies an optimal condition of 7.17 kg load, 18:1 CR, 10% load, 143 NPC and 5.57 L/min of HHO. The addition of Oxyhydrogen enhances combustion and performance while lowering the CO, HC emission with a slight increment of NOx emissions. Sunil Kumar et al.^19^ investigated the engine characteristics using ZrO_2_ NP’s and 1-hexanol additive blended with Moringa oleifera biodiesel blends at 1500 rpm speed. The 90D5MO5H + 25 ppm ZrO_2_ shows 8.63% of higher BTE and lowered BSFC by 46.13% than diesel. The HC emissions for the 100MO+100ppm ZrO_2_ blend shows lowest value of 0.02% while 90D5MO5H+25ppm ZrO_2_ records the minimum CO emissions by 10.1%. Among Gradient Boosting, ELM and RSM methods for modeling, highest prediction accuracy of R^2^ of 0.9604 was achieved. Mohammad Mostafa Namar et al. developed eleven machine learning based regression models to predict the start of combustion in methane fuelled HCCI engines with high speed and accuracy. Both linear and nonlinear approaches were examined where RANSAC and the simple algebraic model improve the prediction accuracy from 89.3% to 98.4%. The comparable accuracy was also achieved by linear models such as ordinary least squares, ridge and Bayesian ridge due to the inherent linear correlation of the combustion parameters. The quick response and high precision of the proposed models make them suitable for engine control applications such as electronic control units. Koten H et al. examined the effects of common rail injector parameters on the performance and emission parameters of a heavy-duty engine using detailed mathematical and statistical analysis of simulation data. The key parameters such as in-cylinder flow, thermodynamic, injection pressure, fuel temperature and intake valve closure timing data were used to train the multiple machine learning models. The developed models accurately predicted the simulation outcomes with drastically reduced computational time which enables accurate optimization. This approach demonstrates considerable time and cost savings which highlights the potential of machine learning for advanced engine design. Sahebalzamani et al. developed machine learning (CFNN and GRNN) models to predict the thermophysical properties of Al_2_O_3_ nano particle enhanced [C_2_mim] [CH_3_SO_3_]-water ionic liquid mixtures. The CFNN-Levenberg-Marquardt model shows an excellent agreement with experiments, achieving exceptionally low AAPRE values for C_p_, thermal conductivity, density, and viscosity. The sensitivity and validation analyses confirmed the dominant influence of nanoparticle concentration and the high reliability of the proposed model for thermal energy applications. Shaisundaram et al. evaluated the combined effect of YSZ-Al_2_O_3_-CeO_2_ thermal carrier coating and CeO_2_ nanoparticles in pumpkin seed biodiesel-diesel blends on CI engine parameters by using the experiments integrated with ANN prediction and RSM based multi-response optimization. The optimized ANN model (R^2^ > 0.99) was accurately predicted SFC, BTE, CO, HC, NOx and smoke and the optimum condition of (B30, 52% load, 50 ppm CeO_2_) yielded a lower SFC and higher BTE and significant emission reductions. Menda et al. investigates the effect of DEE and SWCNT NPs on the performance and emission characteristics of a spirulina platensis microalgae biodiesel-diesel blends in a CRDI engine at different injection pressures. They observed that SVR, RF and DT models were accurately predicted engine responses demonstrating that the potential of ML to reduce the experimental effort and support the optimized engine design. Junhua Li et al. developed a hybrid framework integrating an ensemble and boosting based ML models with statistical design, sensitivity analysis, and SHAP interpretability to predict the performance and emissions of a diesel engine fuelled with microalgae biodiesel blends. The Gradient Boosting achieved the highest accuracy (R^2^ = 0.999 for train, 0.9586 for test, MAPE = 2.58%) with engine load was identified as the most influential parameter.

A comprehensive literature review reveals that biodiesel derived from hemp oil plays a significant role and as a promising alternative to diesel due to renewable and combustible nature. Although several studies have reported the use of biodiesel derived from non-edible feedstocks and the application of nanoparticle additives to enhance the diesel engine performance and emissions, most of these investigations were limited to the single nanoparticle formulations and conventional experimental analysis. The studies on hemp biodiesel blends with nano additives are still very scarce and the combined influence of multiple nano additives on engine parameters has not been comprehensively explored. In addition, recent machine learning approaches for engine studies are focused on the single parameter prediction and do not provide a unified framework for simultaneous multi response modelling by using experimental data from the nano enhanced fuels. The lack of studies on multi nanoparticle enriched hemp biodiesel and integrated AI based multi response prediction and analysis for diesel engine parameters shows a clear research gap. Therefore, there is a strong necessity to develop a strategy that can experimentally evaluate the influence of multi-nanoparticle additives added in the hemp biodiesel used in a diesel engine and accurately predict the different responses of an engine by using advanced machine learning techniques. These approaches can reduce experimental effort, improve prediction capability, and provide a reliable strategy for fuel formulation and engine optimization.

The main objective of this study is to experimentally and computationally investigate the engine parameters of a diesel engine fuelled with hemp biodiesel blend (HMBD20) enriched with multiple nano additives such as TiO_2_, Al_2_O_3_ and MWCNT’s at 50 and 100 ppm concentrations under varying loads. Additionally, three machine learning models such as Decision Tree (DT), Support Vector Machine (SVM) and Artificial Neural Network (ANN) were developed to predict the engine parameters for each response. This study further aims to identify the most effective nano additive formulation and the most reliable machine learning model for predicting and analysing the behaviour of nano enhanced hemp biodiesel used in diesel engines.

## Materials and methods

### Preparation of fuel samples

Hemp raw oil used for this study was procured from the local market at Tirupathi, Andhra Pradesh, India. A total of 1000 ml of hemp seed raw oil was taken and mixed with 200 ml of methanol to function as alcohol agent for maintaining methanol to oil molar ratio as (6:1) along with NaOH (7 g) as the base catalyst also. Initially, hemp oil was heated to 60 °C under a continuous stirring to eliminate moisture then methoxide solution (mixture of methanol and NaOH) was introduced into the preheated oil under magnetic stirring for 90 min to complete transesterification process. Next, the solution was transferred to a funnel and allowed to settle for 24 h to enable phase separation. The upper layer consists of biodiesel and bottom dense layer was glycerol which is removed from the bottom side of the funnel. The prepared biodiesel was blended into diesel fuel to prepare HMBD20 fuel i.e., 20% biodiesel and 80% diesel. The addition of nano particles (TiO_2_, Al_2_O_3_ and MWCNT’s) at a concentration of 50 and 100 ppm in HMBD20 fuel namely HMBD20 + ANP50, HMBD20 + ANP100, HMBD20 + TINP50, HMBD20 + TINP100, HMBD20 + MWCN50 and HMBD20 + MWCN100 fuels. The properties of test fuels were tabulated in Table 1.

**Table 1:** Properties of test fuels.

Type of fuel	Density (kg/m^3^) ASTM D4052	Kinematic viscosity at 40 °C (cst) ASTM D445	Flash point (^0^ C) ASTM D93	Calorific value (KJ/kg) ASTM D240	Cetane number ASTM D613
Diesel	845	2.76	67	44,800	50.5
HMBD20	861	3.26	79	42,750	53.6
HMBD20 + ANP50	867	3.34	78	42,950	54.3
HMBD20 + ANP100	872	3.41	77	43,260	55.2
HMBD20 + TINP50	865	3.31	76	42,910	54.7
HMBD20 + TINP100	869	3.39	75	43,320	55.4
HMBD20 + MWCN50	863	3.36	74	43,060	55.2
HMBD20 + MWCN100	867	3.44	73	43,410	54.8

### FTIR spectra of hemp biodiesel

**Fig. 1:**
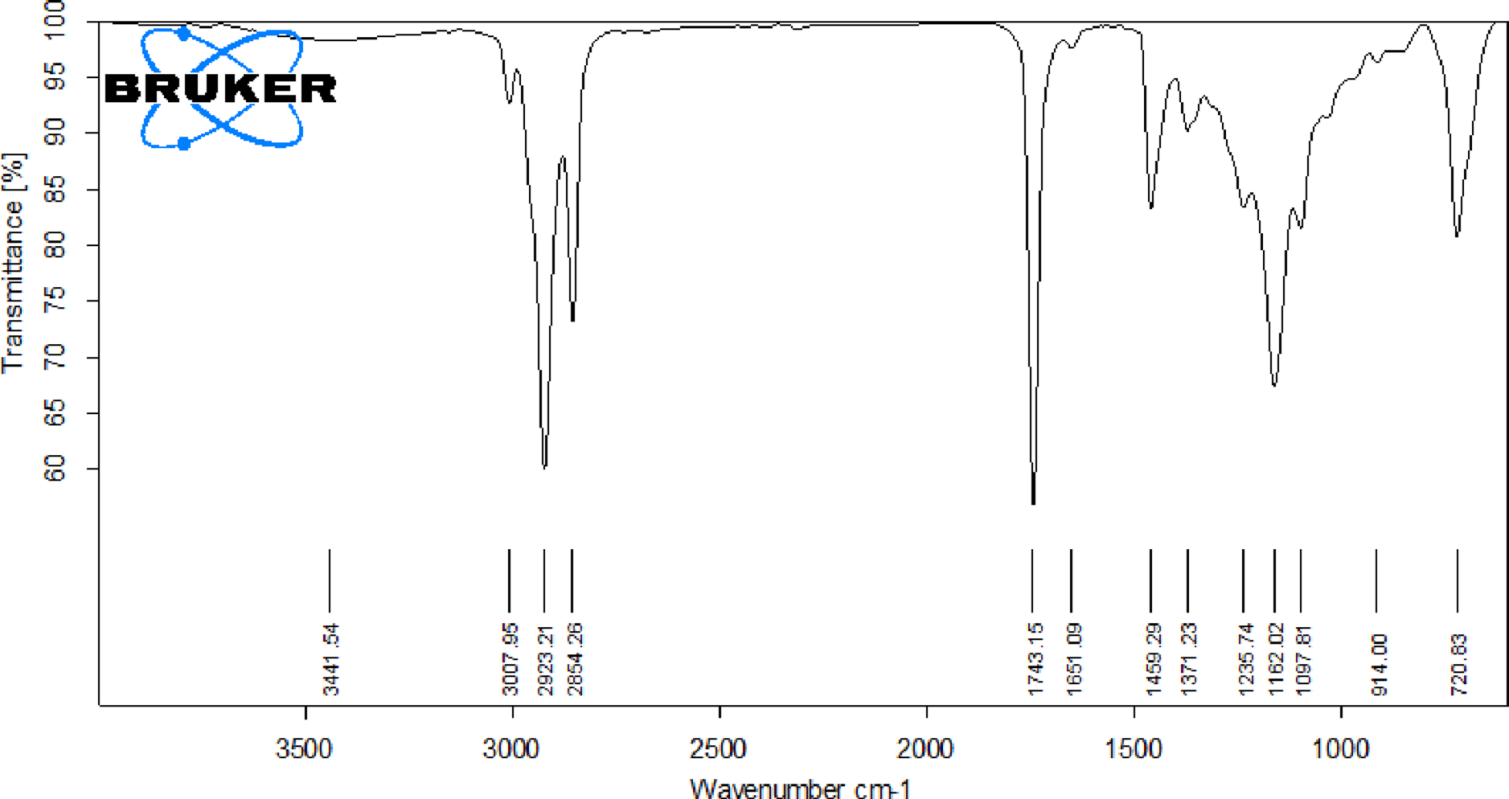
FTIR spectrum of hemp biodiesel.

The FTIR spectrum of hemp biodiesel was shown in Fig. 1 reveals a prominent absorption peak indicates the functional groups of fatty acid methyl esters. The broad peak observed at 3441.54 cm^− 1^ shows O-H stretching vibrations, peaks at 3007.95, 2923.21, 2854.29 cm^− 1^ were attributed to symmetric and asymmetric stretching vibrations of CH_2_ and CH_3_ groups confirm the hydrocarbon chains present in the biodiesel. A strong absorption peak at 1743.15 cm^− 1^ is allotted with the C = O stretching vibration of ester groups is an indicative of methyl ester confirms the transesterification process. The peak at 1601.09 cm^− 1^ represents the C = C stretching vibrations due to the occurrence of unsaturated carbon chains in the biodiesel. The bands at 1459.29 cm^− 1^ and 1375.74 cm^− 1^ correspond to the bending vibrations of CH_2_ and CH_3_ groups, respectively. The peaks at 1235.74 cm^− 1^, 1102.72 cm^− 1^ and 1087.92 cm^− 1^ are an indicative of C-O stretching vibrations related to the formation of ester linkages. The C-H bending of alkenes at the absorption band observed at 914.00 cm^− 1^ endorses the retention of some unsaturation in the alkyl chains. The band at 720.83 cm^− 1^ corresponds to the rocking vibration of long CH_2_ chains.

### GCMS analysis of hemp biodiesel

**Fig. 2:**
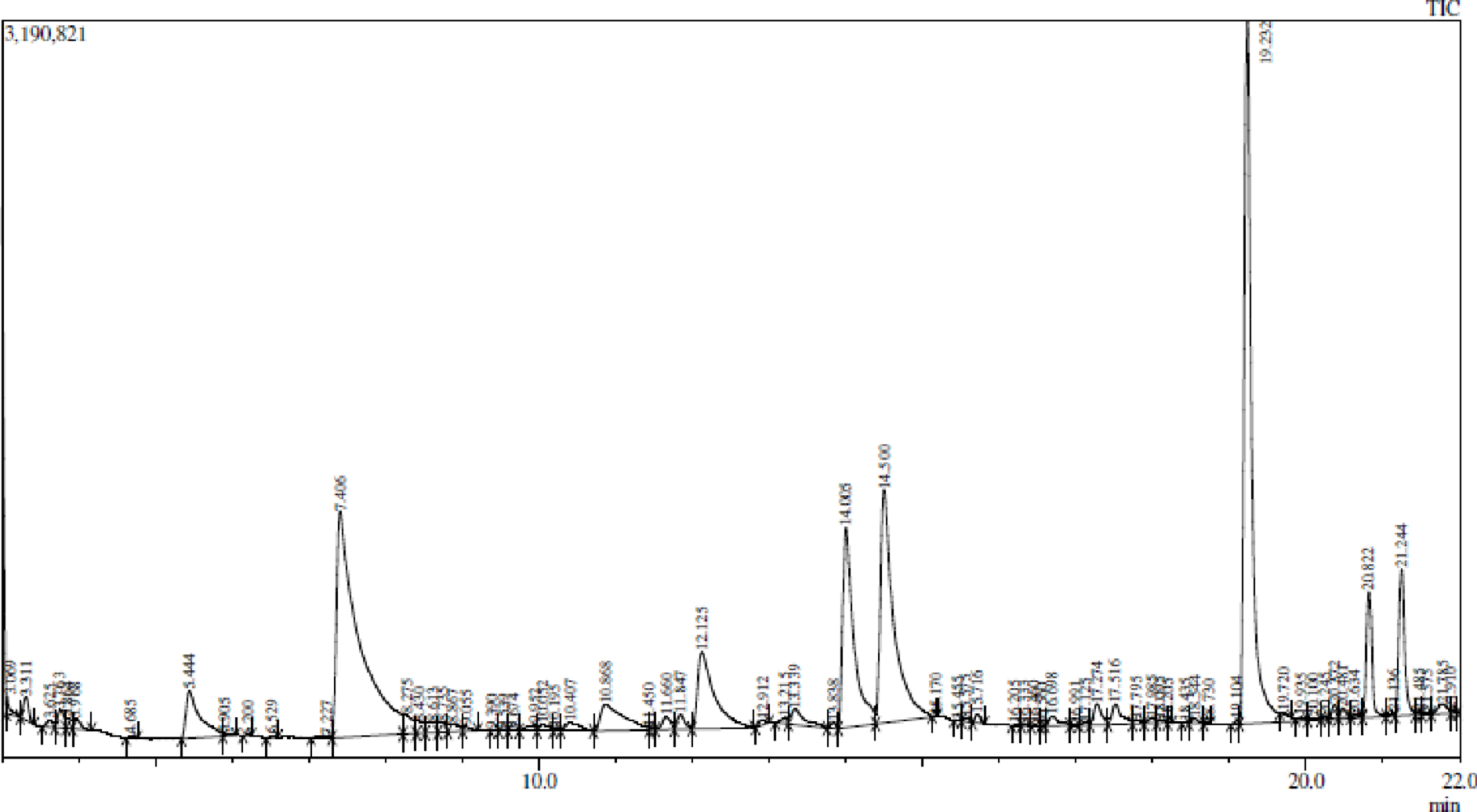
GCMS of hemp biodiesel.

The gas chromatographic profile of hemp biodiesel shown in Fig. 2 reveals a distribution of fatty acid methyl esters along with long-chain hydrocarbons, aldehydes and oxygenated components typically related to transesterification and subsequent oxidation of unsaturated esters. The saturated components such as methyl heptanoate (C7:0 ME), pentadecanoic acid (C15:0), Octadecanoic acid (stearic acid, C18:0) and hexadecanoic acid methyl ester were confirmed and contributed to greater oxidative stability. The unsaturated esters quantity was dominated by polyunsaturated species, particularly octadecadienoic acid accompanied by minor unsaturated hydrocarbons such as pentadecane and tetradecen-1-ol. It reflects the inherent lipid composition of hemp biodiesel characterized by the polyunsaturated fatty acids causes an auto oxidation and peroxide formation. The long chain hydrocarbons like dodecane, tridecane, tetradecane and heptadecane were also identified as intrinsic components and their presence slightly enhanced the energy density with negligible contribution of lubricity properties. The minor compounds such as isothiocyanate, dimethyl octanol and benzoic acid were also observed but not directly contributed to fuel properties. Table 2 shows the GCMS profile of Hemp biodiesel.

**Table 2:** GCMS Profile of hemp biodiesel.

Sl.NO.	RT (min)	Compound (tentative ID)	Class
1	5.44	Allyl isothiocyanate	Oxygenated compound
2	7.23	11-(2-Cyclopenten-1-yl) un decanoic acid	Unsaturated FA derivative
3	10.41	Methyl heptanoate (C7:0 ME)	Short-chain FAME
4	11.66	3,7-Dimethyl-7-octen-1-ol	Long-chain alcohol
5	12.12	Benzoic acid, 2-hydroxy-, methyl ester	Aromatic ester
6	13.34	E-2-Decenal	Unsaturated aldehyde
7	13.84	Tridecane	Hydrocarbon (C13)
8	14.00–14.50	2,4-Decadienal (E, E)	Unsaturated aldehyde
9	15.71	Tetradecane (C14)	Hydrocarbon
10	17.27	cis-7-Tetradecen-1-ol	Long-chain alcohol
11	17.52	Dodecane (C12)	Hydrocarbon
12	17.79	Heptadecane (C17)	Hydrocarbon
13	19.94	Octadecanoic acid (C18:0, stearic acid)	Saturated FA
14	20.1	Pentadecanoic acid (C15:0)	Saturated FA
15	20.37	Cycloundecene (Z)	Unsaturated hydrocarbon
16	20.48	1-Pentadecene (C15:1)	Unsaturated alkene
17	20.82	Heptadecane (C17:0 hydrocarbon)	Saturated hydrocarbon
18	21.24	Hexadecanoic acid, 15-methyl-, methyl ester (iso-C17:0 ME)	Saturated FAME
19	21.79	9,12-Octadecadienoic acid (Z, Z)- (linoleic acid, C18:2)	Polyunsaturated FA
20	21.91	2-Azaspiro [4.4] non-2-ylmethanamine	N-heterocyclic compound

### Characterization of nanoparticles

#### SEM and XRD of Al_2_O_3_ NP’s

**Fig. 3:**
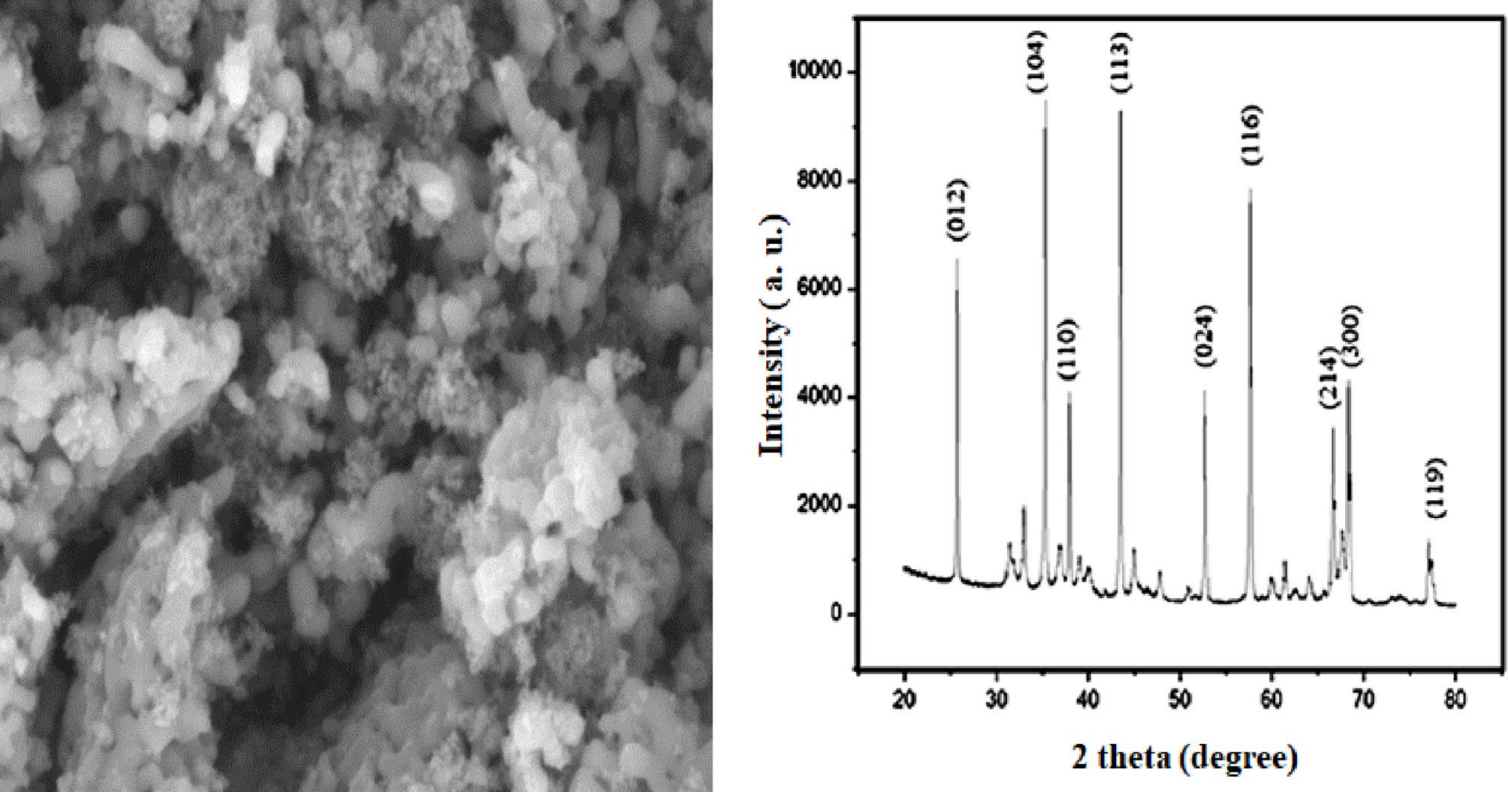
SEM & XRD of Al_2_O_3_ NP’s.

Figure 3 shows the SEM and XRD images of Al_2_O_3_ Np’s. The SEM image was examined the surface morphology and particles were distributed as quasi-spherical tendency which exhibits as a high surface energy due to van der Waals forces between particles. These morphological characteristics were causes to catalytic behavior because of high surface to volume ratios. The EDS analysis of NP detected carbon (C), oxygen (O), and aluminum (Al) with weight percentages of 7.10%, 40.18%, 52.72%, respectively. The elemental composition supports the formation of Al_2_O_3_ NPs with a dominant quantity of aluminum and oxygen components and validated with phase purity, consistent with the XRD results. The crystalline phases of NP’s were identified by XRD image by using distinct peaks observed at 2 theta values of approximately 25.6^0^ (012), 35.1^0^ (104), 37.7^0^ (110), 43.3^0^ (113), 52.6^0^ (024), 57.6^0^ (116), 66.6^0^ (214), 68.2^0^ (300), 77.0^0^ (119). The absence of secondary or impurity peaks indicates high phase purity and sharpness of the peaks confirms the crystalline nature of NPs. The average crystallite size of the NP’s was estimated as 42.10 to 56.50 nm by Screrrer equation.

#### SEM and XRD of MWCNT’s

**Fig. 4:**
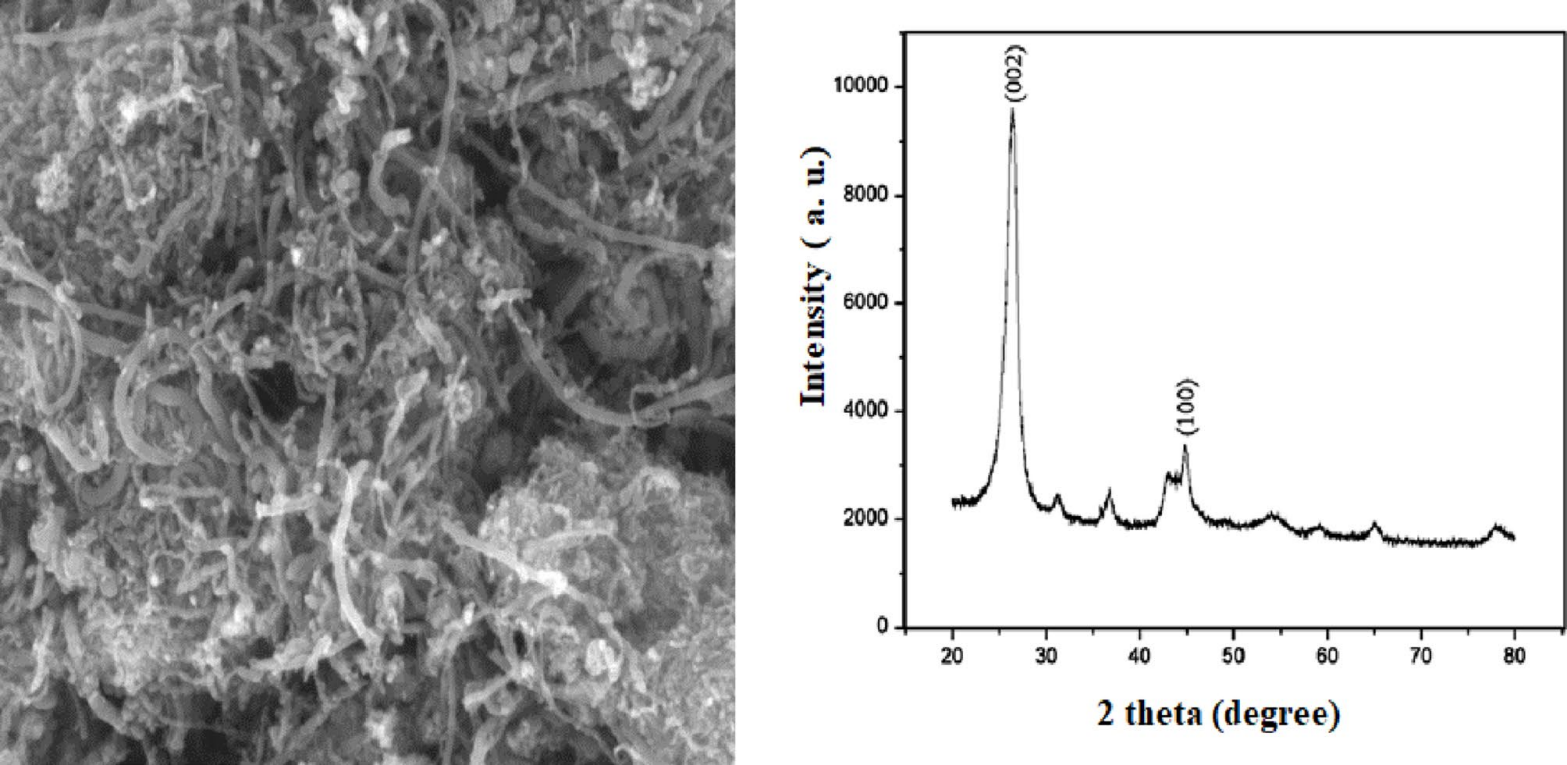
SEM & XRD of MWCNT’s.

Figure 4 shows the SEM and XRD images of MWCNT’s. The SEM image reveals the tubular structures with uniform diameters which confirm the multi walled carbon nanotubes. The dense framework of nano tubes in the image is due to van der Waals interactions between adjacent tubes and some agglomeration was also observed. The nanotube structure provides a high surface-to-volume ratio which is suitable for energy and combustion applications. The EDS of MWCNT’s reveals that, the sample contains elements of carbon (C), oxygen (O), iron (Fe) with weight percentages of 95.83%, 3.81% and 0.36%, respectively. The major contributor is carbon, with minor content of oxygen indicates the graphitic structure of multi walled nano tubes with high purity. The crystalline structure of MWCNT’s was analyzed by XRD using the diffraction peaks observed at 26.1^0^ (002), 43.2^0^ (100) confirms with the standard JCPDS values. The sharp and intense reflection at (002) reveals the high degree of graphitization and weak peak at (100) is due to plane periodicity of the graphitic lattice.

#### SEM and XRD of TiO_2_ NP’s

**Fig. 5:**
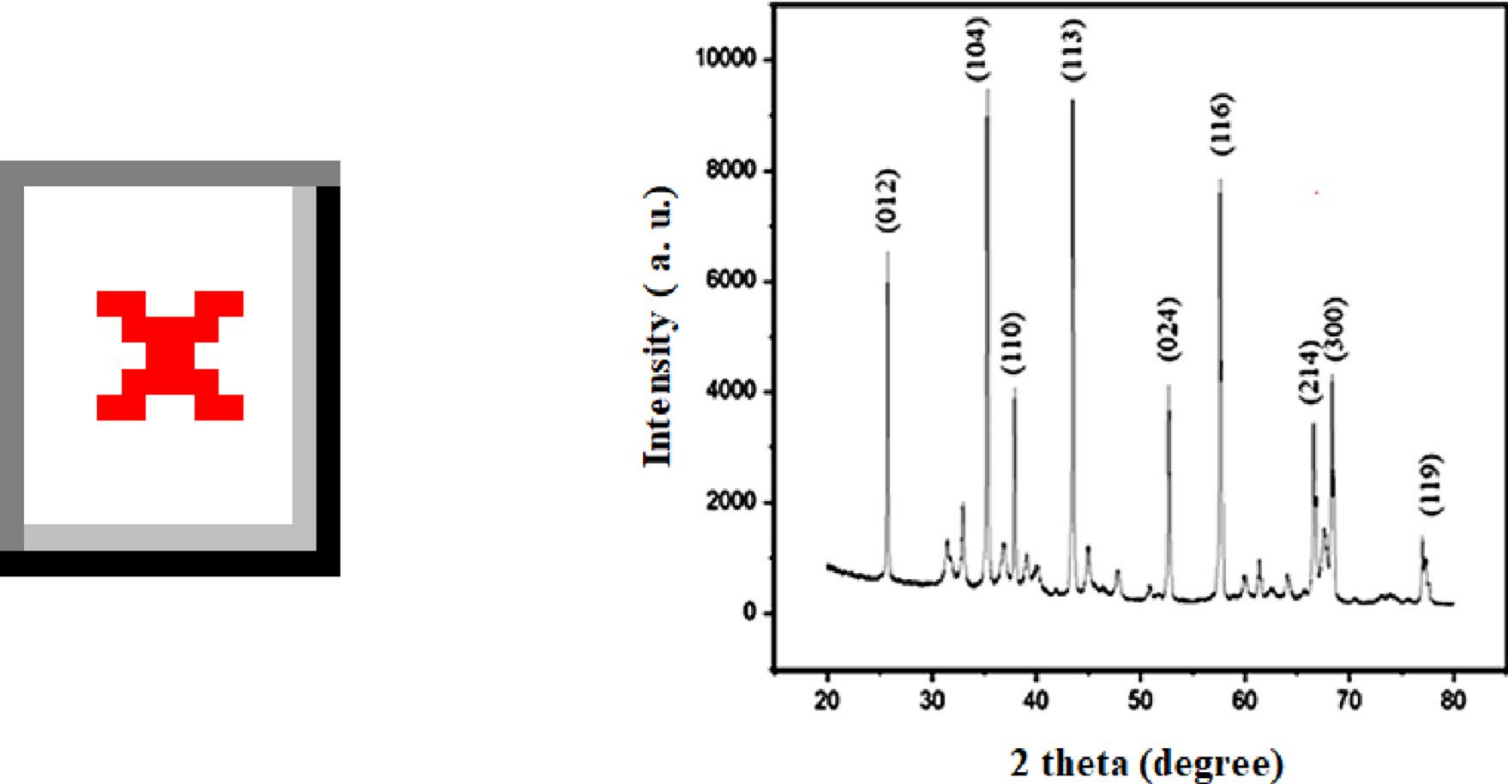
SEM & XRD of TiO_2_ NP’s.

**Fig. 6:**
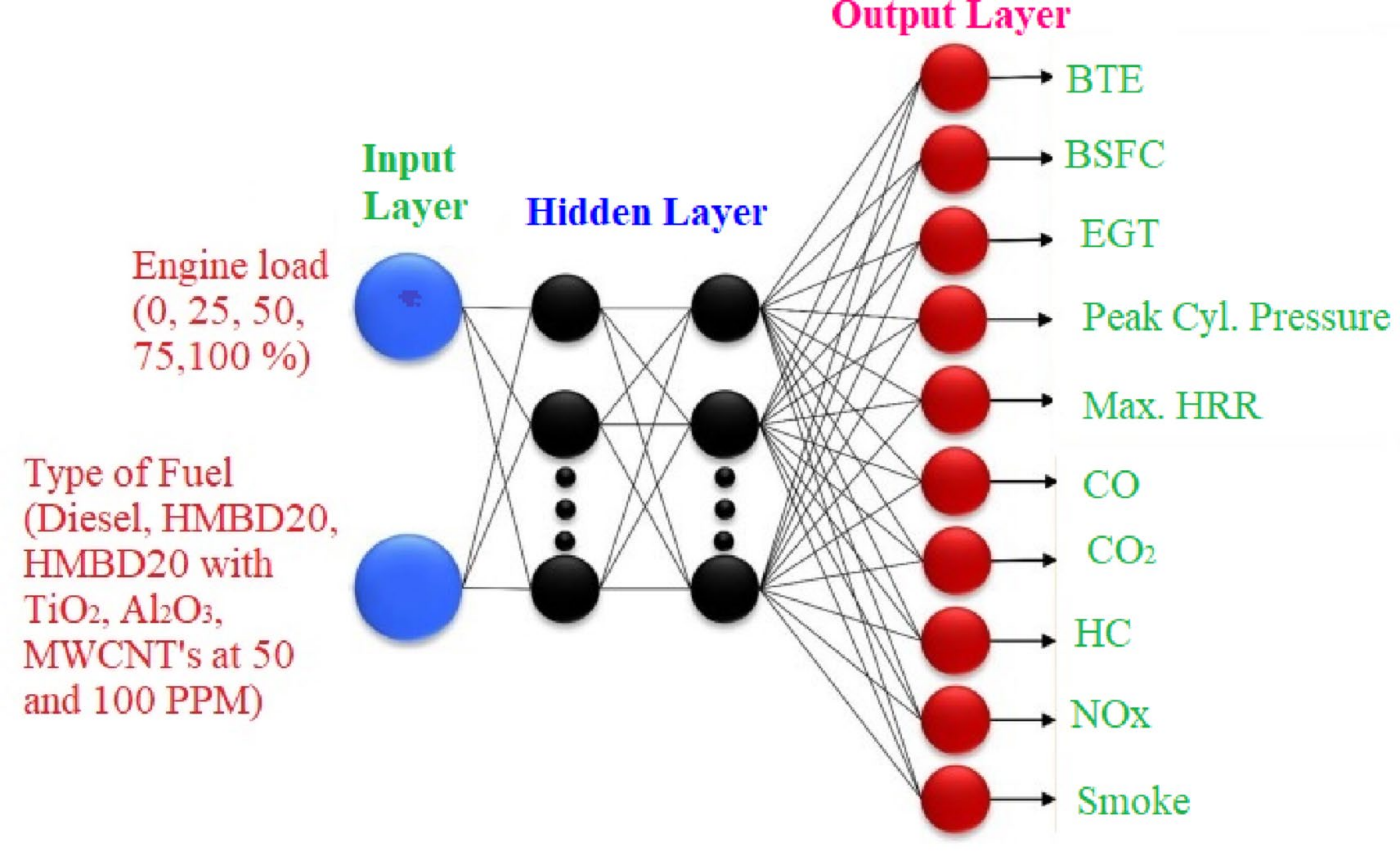
ANN architecture reproduced from^41^.

Figure 5 shows the SEM & XRD images of TiO_2_ NPs. The SEM image of TiO_2_ NPs contains spherical shape with high surface energy and uniform distribution of fine particles. In various regions, porous structures and clusters were also observed which enhances the surface to volume ratio makes it suitable for catalytic based applications. The EDS of the TiO_2_ NP confirms the composition of titanium, oxygen, and carbon with weight percentages of 57.08%, 39.54% and 3.38%, respectively. The major contributors of Ti and O can lead to the formation of titanium dioxide (TiO_2_) with high purity. The XRD pattern displays the sharp and intense diffraction peaks at 25.3^0^ (101), 37.8^0^ (004), 48.0^0^ (200), 53.9^0^ (105), 55.1^0^ (211), 62.7^0^ (204), 68.8^0^ (116), 70.3^0^ (220) and 75.1^0^ (215) matches standard planes of the anatase phase of TiO_2_ NPs. The absence of impurity peaks confirmed the high phase of purity of nano particles. The average crystallite size was estimated to be 45–62 nm by using Scherrer equation and closely matches with the morphology of SEM which confirms the well-defined crystalline structure (Fig. 6).

## Experimental methodology

### Engine setup and procedure

**Fig. 7:**
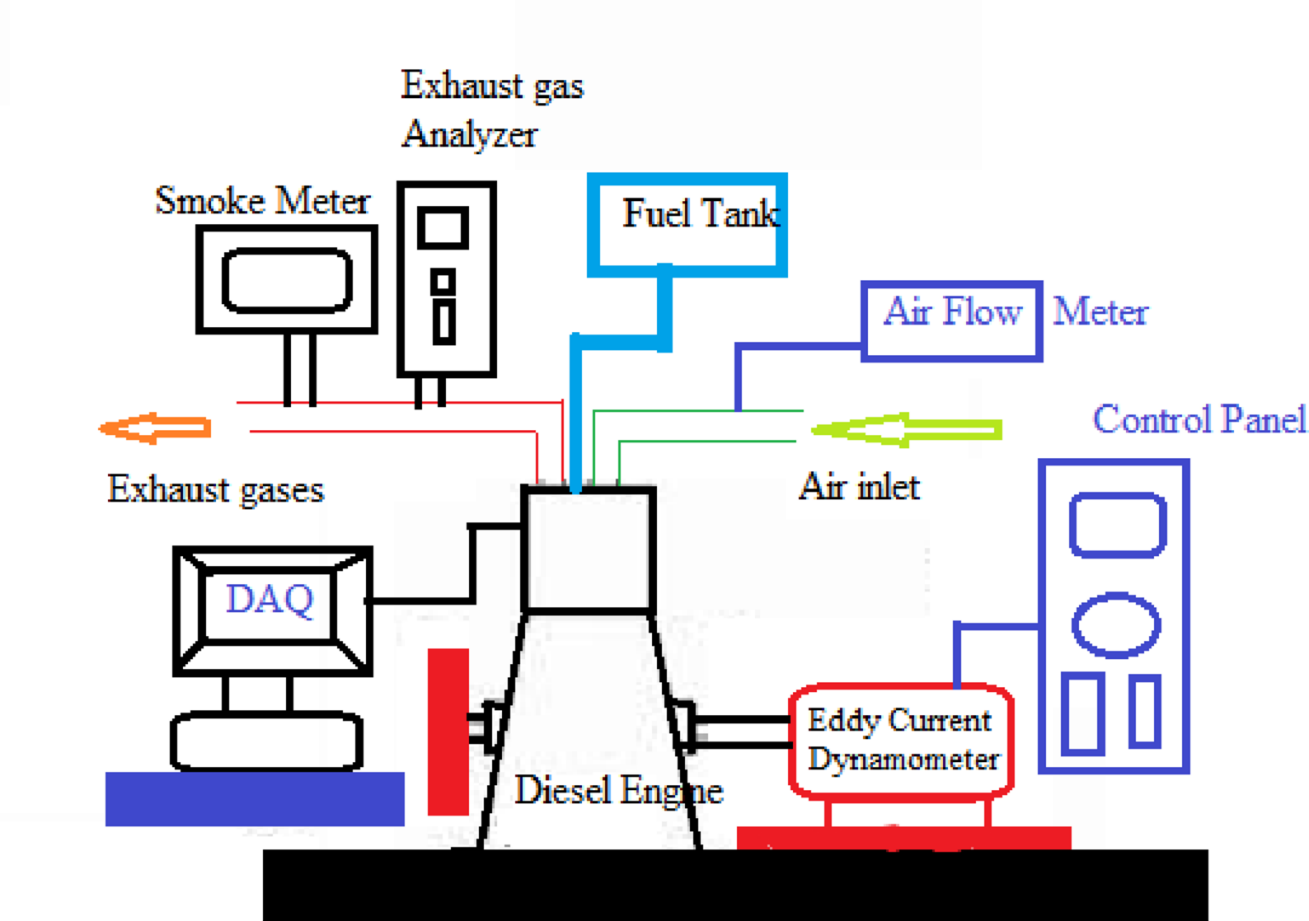
Schematic diagram of test engine.

The engine tests were conducted on single cylinder, Kirloskar make 4-stroke, water cooled DI diesel engine with a rated speed of 1500 rpm. The engine was coupled with eddy current dynamometer for applying loads. In cylinder pressure (up to 250 bar) was measured by a piezoelectric pressure transducer which is mounted on engine cylinder head. K-type thermocouples with good precision were used to determine temperatures at various locations. The engine has specifications of 87.5 mm of bore diameter, 110 mm of stroke length, 17.5:1 of CR and rated power of 5.4 kW. The engine speed was monitored using a non-contact digital tachometer with ± 10 rpm accuracy. A LAB-VIEW based software was adopted for synchronizing engine input parameters by using DAQ system for estimating the engine output parameters. The engine tests initially conducted with diesel fuel then with HMBD20 fuel next, using nano additive fuel samples in various loads to estimate the engine characteristics. The AVL 444 five gas analyzer and AVL 437 C smoke meter were used to assess the engine pollutants. The Schematic diagram of test engine was depicted in Fig. 7. The engine specification with accessories was tabulated in Table 3.

**Table 3:** Engine specifications along with measuring instruments.

Engine parameter	Specifications
Engine Make and Type	TV1 model Kirloskar make, 4-stroke, Single cylinder DI Diesel engine
Bore and stroke	87.5 × 110 mm
FIT & FIP	23 ^0^bTDC & 220 bar
Rated Power & Speed	5.2 kW & 1500 rpm
Exhaust gas Analyzer	AVL DiGas 444 N (NOx, CO, HC, CO_2_)
Smoke Meter	AVL 437 C (Smoke emissions)
Crank Angle	Optical encoder

### Uncertainty analysis

To ensure the accuracy and reliability of the experimental results, an uncertainty analysis was performed based on the Root Sum of Square (RSS) method. It is widely used when multiple independent uncertainties arose during measuring of an instrument while testing. This approach provides a statistically robust method for estimating overall uncertainty by considering the errors of individual instruments. The diesel engine parameters, ranges along with their units were tabulated in Table 4. The following expression is used to compute the overall certainty of the experiments. The overall uncertainty was measured by using the following equation considering individual parameters uncertainty of BP (0.9), Speed (1.2), BTE (0.75), BSFC (1.05), EGT (0.92), CO (0.85), HC (1.09), NOx (1.05), and smoke (0.95) and estimated as 2.94%.

**Table 4:** Diesel engine parameters, ranges along with their units.

Parameter	Range	Unit
Engine Load	0–100	%
Engine speed	1500 (Fixed)	RPM
Brake Power	0–5.2.2	kW
Exhaust gas temperature	30–550	^0^C
Injection pressure	200–220	bar
Nano particle Concentration	50–100	ppm
Cylinder pressure	10–85	bar
Heat release rate	10–90	J/^0^ CA
Carbon monoxide	0.05–0.34	% Vol
Hydrocarbons	17–70	ppm
Nitrogen oxides	120–2500	ppm
Smoke opacity	2.5–75.0	%

.

$$U_p=\:\sqrt{{\left(\frac{\partial\:P}{\partial\:{x}_{1}}\:{U}_{x1}\right)}^{2}+\:{\left(\frac{\partial\:P}{\partial\:{x}_{2}}\:{U}_{x2}\right)}^{2}+\dots\:+\:{\left(\frac{\partial\:P}{\partial\:{x}_{n}}\:{U}_{xn}\right)}^{2}}$$

Where U_p_ = Uncertainty of all parameters.

U_X1_, U_X2,_ ……. Uxn are the uncertainties of each parameter.

### Machine learning techniques

The ML techniques were adopted to model the nonlinear relationships between input parameters and output responses to predict the performance and emissions of an engine. To avoid overfitting and ensure the reliable model generalization, the dataset was divided into training and testing subsets, and the model performance was primarily evaluated based on the testing data. In the Decision Tree model, the tree depth and minimum number of samples per leaf were optimized to prevent the excessive growth of the tree. In SVM model, appropriate kernel parameters and regularization were selected to control the model complexity and to maintain the structural risk minimization principle. For the ANN model, the number of hidden neurons and training iterations were carefully tuned to avoid over parameterization. The close agreement between the training and testing error values indicates that the developed models did not suffer from overfitting and were able to generalize the underlying nonlinear relationship between the input and output parameters.

Decision tree is used for regression and classification purposes in building flow chart like tree structures. Decision tree models split the dataset recursively into subsets on input variables to minimize impurity and this model aims to reduce the mean squared error (MSE) in each partition. The DT model assumes that the relationship between input parameters and engine responses can be represented through a hierarchical rule-based partitioning of the dataset into a homogeneous subset. It does not require any prior assumption on data distribution or linearity. The most influential features were identified based on their ability to reduce the prediction error during recursive splitting. The split is considered to minimize the weighted sum of MSE of the sub node.

 

$$MSE=\:\frac{1}{n}\:\sum_{i=1}^{n}({Y}_{pi}-\:{Y}_{ai})^{2}$$

$$\:\underset{{\:\:\:\:\:\:\:\:\:\:\:\:\:\:\:\:\:\:\:\:}J,S}{\mathrm{Split}=\mathrm{min}}\left[\frac{{n}_{L}}{n}\:\sum\:_{i\:\in\:L}{\left({y}_{i}-{\stackrel{-}{y}}_{L}\right)}^{2\:}+\:\frac{{n}_{R}}{n}\:\sum\:_{i\:\in\:R}{\left({y}_{i}-{\stackrel{-}{y}}_{R}\right)}^{2\:}\:\right]$$

Where Y_pi_ and Y_a.i._ are the predicted and actual values, y_i_ is the output variable, $\:{\stackrel{-}{y}}_{L}$and $\:{\stackrel{-}{y}}_{R}$ are the mean output variables of the left and right subsets, $\:{n}_{L}$, $\:{n}_{R}$ and * n* represents the samples in each node and total samples respectively, J and S represents the split error and split position, split is the process of dividing the dataset into two smaller groups based on a condition on an input variable to reduce the prediction error.

Support Vector Machines are kernel-based learning algorithms that interrelate input variables into higher dimensional space using kernel functions. The optimal hyperplane in the SVM model was developed to minimize the prediction error for complex and nonlinear behavior in the combustion and performance of diesel engine data. The usage of kernel functions meant for accurate modelling of input and output variables relationships associated with varying load conditions and nano additive concentrations. The SVM model assumes that the nonlinear interaction between the inputs and outputs can be mapped into a higher dimensional feature space using a suitable kernel function. It considers the existence of a global optimal regression hyperplane that minimizes the structural risk and improves the generalization. The selected kernel is assumed to be capable of capturing complex engine behavior. SVM model developed a function that estimates the target variable within a margin of tolerance.

$$\:F\:\left(x\right)=\sum_{i=1}^{n}\left({a}_{i}-\:{{a}_{i}}^{*}\right)K(x_i,x)+b$$

Here F(x) is the predicted output for a new output x, $\:{\alpha\:}_{i}$, $\:{{\alpha\:}_{i}}^{*}$ are Lagrange multipliers, K (x_i_, x) is the kernel function and b are the bias term.

Artificial Neural Network is a powerful modelling tool that identifying complex and nonlinear relationships between input and output variables especially suitable for diesel engine performance and emissions characteristics at various loads. ANN consists of artificial neurons organized in layers such as input layer, output layer and one or more hidden layers containing weighted inputs and biases to minimize the prediction error. The ANN model assumes that the engine parameters followed a nonlinear input-output relationship that can be approximated by using interconnected neurons and hidden layers. It is assumed that the training dataset is sufficient for learning the patterns through a backpropagation. The chosen network architecture was considered to be adequate to generalize the system behaviour. The following formulas were used for estimating performance metrics. The ANN Architecture was shown in Fig. 6.

$$R^{2}=\:1-\:\sum_{i=1}^{n}\left(\frac{{\left({y}_{pi\:}-{y}_{ai}\right)}^{2}}{{\left({y}_{ai}-\:{y}_{m}\right)}^{2}}\right)$$

$$MAE=\:\:\frac{1}{n}\:\sum_{i=1}^{n}\left|{y}_{ai}-\:{y}_{pi}\right|$$

$$RMSE =\:\sqrt{\sum_{i=1}^{n}\frac{{\left({y}_{pi}\:-\:{y}_{ai}\right)}^{2}}{n}}$$

Where R^2^ is the Coefficient of determination, y_pi_, y_a.i._ and y_m_ are the predicted values, actual experimental values and mean of actual values respectively, n is the number of data points. MAE means absolute error means the difference of experimental and predicted values. RMSE is the root that means square error which provides the standard deviation of prediction errors.

The DT, SVM and ANN models were selected because they represent three fundamentally different learning strategies such as rule based learning, kernel-based learning and neuron based nonlinear mapping allows a comprehensive evaluation of prediction capability for nano additive fuel samples used for diesel engine operation. Compared to conventional regression models these techniques can effectively capture the complex and highly nonlinear interactions between the fuel samples, engine load and performance and emission parameters.

## Results and discussions

This chapter presents a detailed analysis of the experimental results of a diesel engine using different fuel samples namely, diesel, HMBD20, HMBD20 fuel with 50 and 100 ppm concentration of TiO_2_, Al_2_O_3_ and MWCNT’s on the performance and emission characteristics at different loads.

### Brake Thermal Efficiency (BTE)

**Fig. 8:**
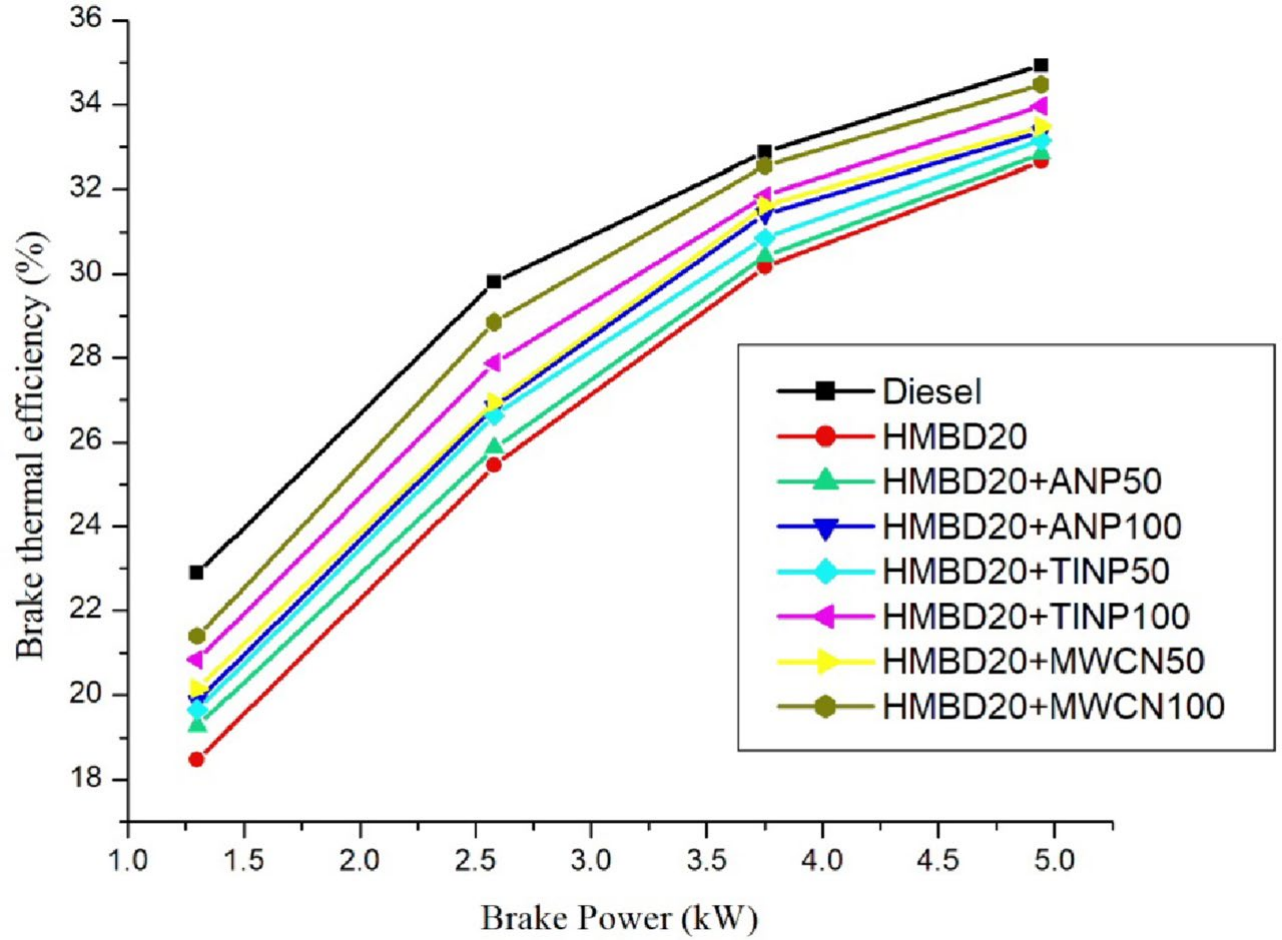
Variation of BTE with BP for all test fuels.

Figure 8 presents the variations of BTE for all fuels. The consistent increase in BTE was noted with rising load due to improved volumetric efficiency. The lowest calorific value of biodiesel, HMBD20 fuel shows 6.98% reduced BTE than diesel at peak load (4.94 kW). The addition of Al_2_O_3_, TiO_2_ and MWCNT’s to the HMBD20 fuel enhances the BTE across all loads. The inclusion of NP possesses a high surface area and thermal conductivity which promotes the faster evaporation of fuel droplets and shortens the ignition delay and intensifies the premixed combustion phase. The HMBD20 + MWCN100 fuel exhibits highest increment (5.60%) than HMBD20 fuel at full load because of the catalytic nature of MWCNTs. The BTE for HMBD20 + ANP50, HMBD20 + TINP50 and HMBD20 + MWCN50 fuels increased by 0.58%, 1.53% and 2.54% respectively at peak load (4.94 kW). It is also observed that HMBD20 + ANP100, HMBD20 + TINP100 fuels also shows an enhancement of 2.17% and 4.01% respectively compared to HMBD20 fuel due to increased fuel-air mixing causes to complete combustion of fuel^21^ The superior performance of MWCNT doped fuel also suggests its potential for optimizing the biodiesel formulations for high efficiency diesel engine operation.

### Brake Specific Fuel Consumption (BSFC)

**Fig. 9:**
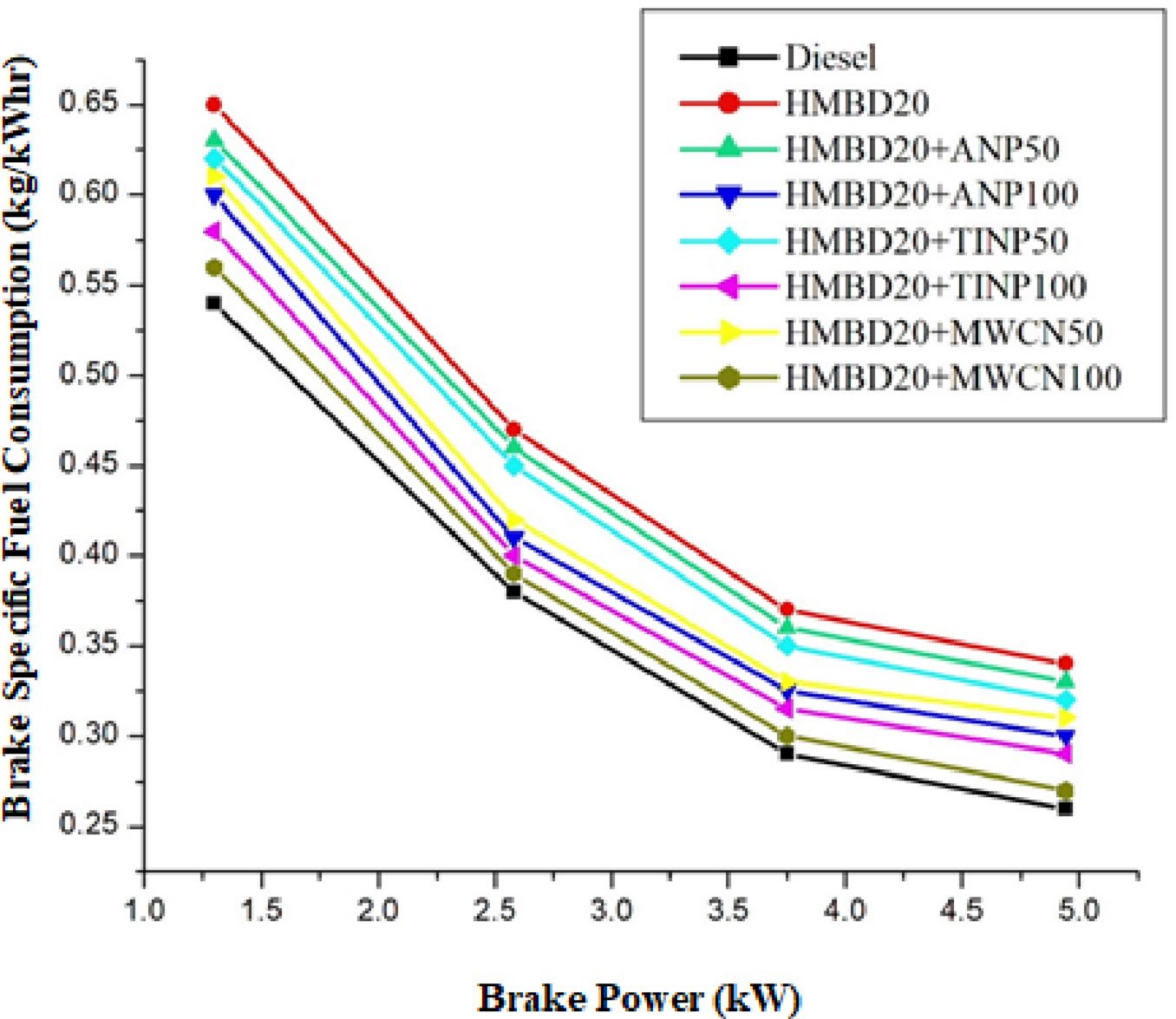
Variation of BSFC with BP for all test fuels.

Figure 9 shows the variation of BSFC for all fuels. The BSFC for HMBD20 fuel was higher than diesel at all load conditions. This is due to lower volatility, higher viscosity and longer ignition delay of biodiesel which results in inferior atomization and slower combustion. The HMBD20 + ANP50, HMBD20 + TINP50 and HMBD20 + MWCN50 fuels show reduced BSFC by 2.94%, 5.88% and 8.82% compared to HMBD20 fuel at peak load. It is also observed that HMBD20 + ANP100, HMBD20 + TINP100 and HMBD20 + MWCN100 fuels diminish BSFC by 11.76%, 14.7% and 18.58% than HMBD20 fuel. The addition of nano particles such as TiO_2_ and Al_2_O_3_ causes improved atomization of fuel, micro explosion of fuel droplets leads to lower consumption of fuel^31^. The presence of MWCNTs in fuel accelerates the heat transfer rate that allows the ignition and flame propagation properties that leads to reduction of fuel consumption per unit BP^22^. The reduction of BSFC relates to the lower specific fuel consumption and improved fuel economy for diesel engines operating with biodiesel blends which are beneficial for stationary power generation and transport applications.

### Exhaust Gas Temperature (EGT)

**Fig. 10:**
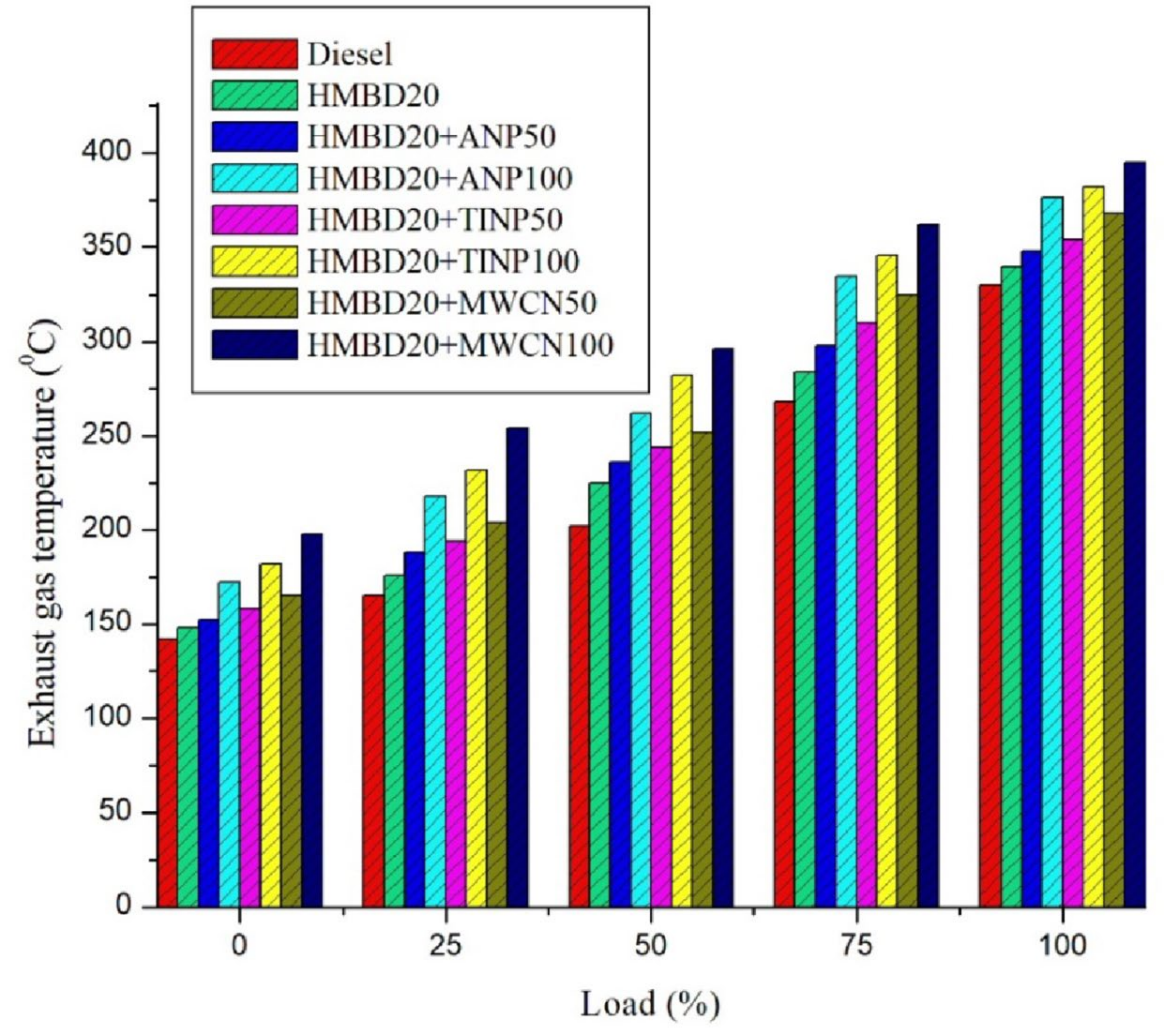
Variation of EGT with load.

Figure 10 depicts the variations of EGT with load for test fuels. The rise in EGT was noted with an increase in load for all fuels because of enhanced fuel injection rate and in-cylinder pressure at higher loads. A slight improvement in EGT was observed for biodiesel blend (HMBD20) than diesel at all loads. This can be attributed to the oxygenated nature of biodiesel that promotes complete combustion and higher cylinder temperatures. At full load, the EGT was higher for the HMBD20 + MWCN50 and HMBD20 + MWCN100 fuels by 8.23% and 16.17% compared to HMBD20 fuel due to higher thermal conductivity and improved flame propagation of CNT’s results in greater exhaust temperatures. The addition of TiO_2_ and Alumina NP’s to HMBD20 fuel at 50 and 100 ppm also enhances the EGT by 2.35, 9.67, 4.11 and 11.65% than HMBD20 fuel because these NP’s contains oxygen content which reduces the ignition delay and increases combustion chamber temperature^35^. The increase in EGT indicates the improved combustion efficiency and more effective energy release inside the cylinder, which is consistent with the enhancement of BTE and reduction of BSFC.

### Peak Cylinder Pressure (PCP)

**Fig. 11:**
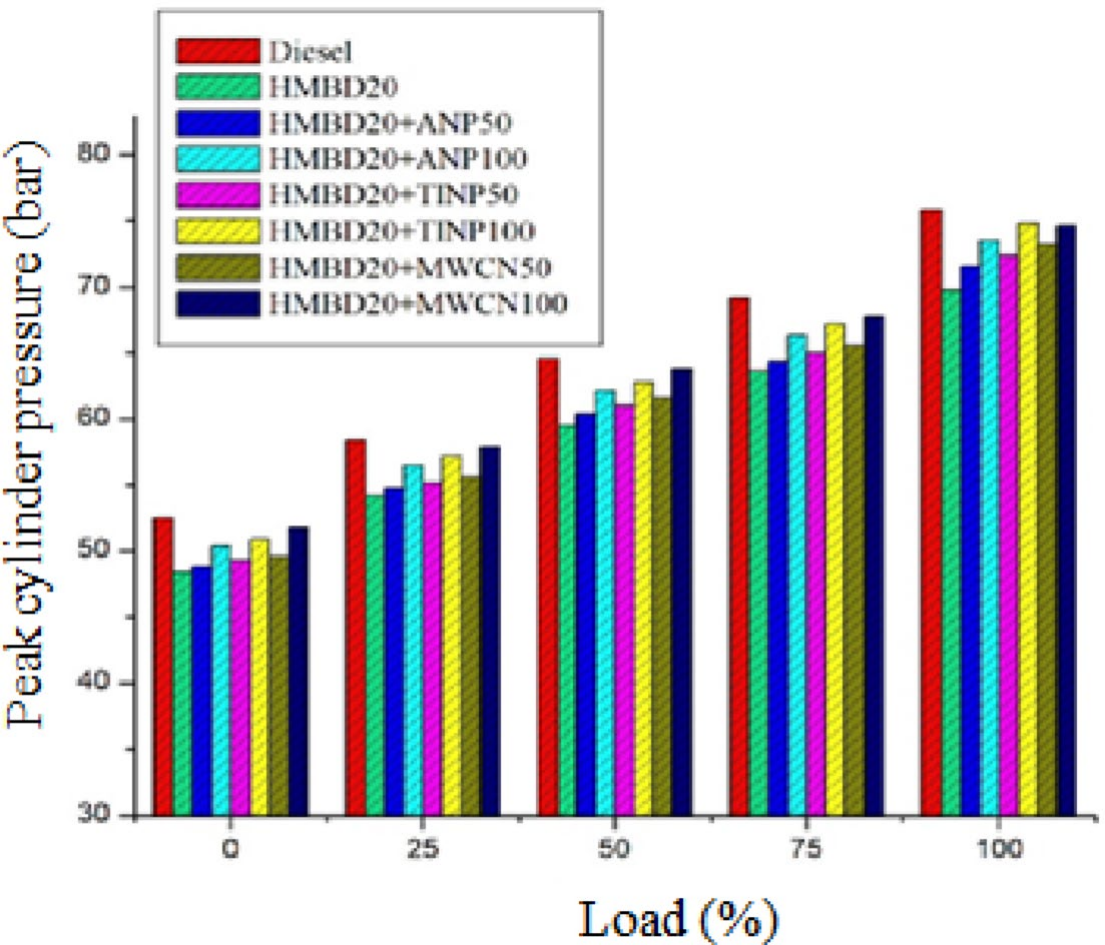
Variation of Peak Cylinder Pressure (PCP) with load for all test fuels.

Figure 11 depicts the peak cylinder pressure for test fuels at various loads. Peak cylinder pressure reflects the combustion intensity within cylinder to effects engine efficiency and emissions. At full load diesel exhibits the highest peak cylinder pressure (78 bar) followed by HMBD20 + MWCN100 (76.8 bar) and HMBD20 + TINP100 (76.2) fuels. The HMBD20 fuel shows lowest peak cyl. pressure than diesel due to lower cetane number and greater density of biodiesel causes retardation of ignition in diffused combustion stage. A significant improvement was observed for the Titania and Alumina NP’s blended HMBD20 fuel because of oxygen content and acts as a combustion catalyst which helps in generating of high pressure and temperatures^30^. The increased surface area of MWCNT’s enables the better atomization of the fuel which leads to higher pressure inside the cylinder of an engine^32^. The increase in peak cylinder pressure with NP addition indicates more efficient conversion of chemical energy into useful work also the higher in-cylinder pressure influences the engine loading. The results suggest that nano-enhanced HMBD20 fuel can achieve combustion characteristics comparable to diesel at high load making it a promising alternative fuel for high efficiency diesel engine applications without any modification.

### Maximum HRR (MHRR)

**Fig. 12:**
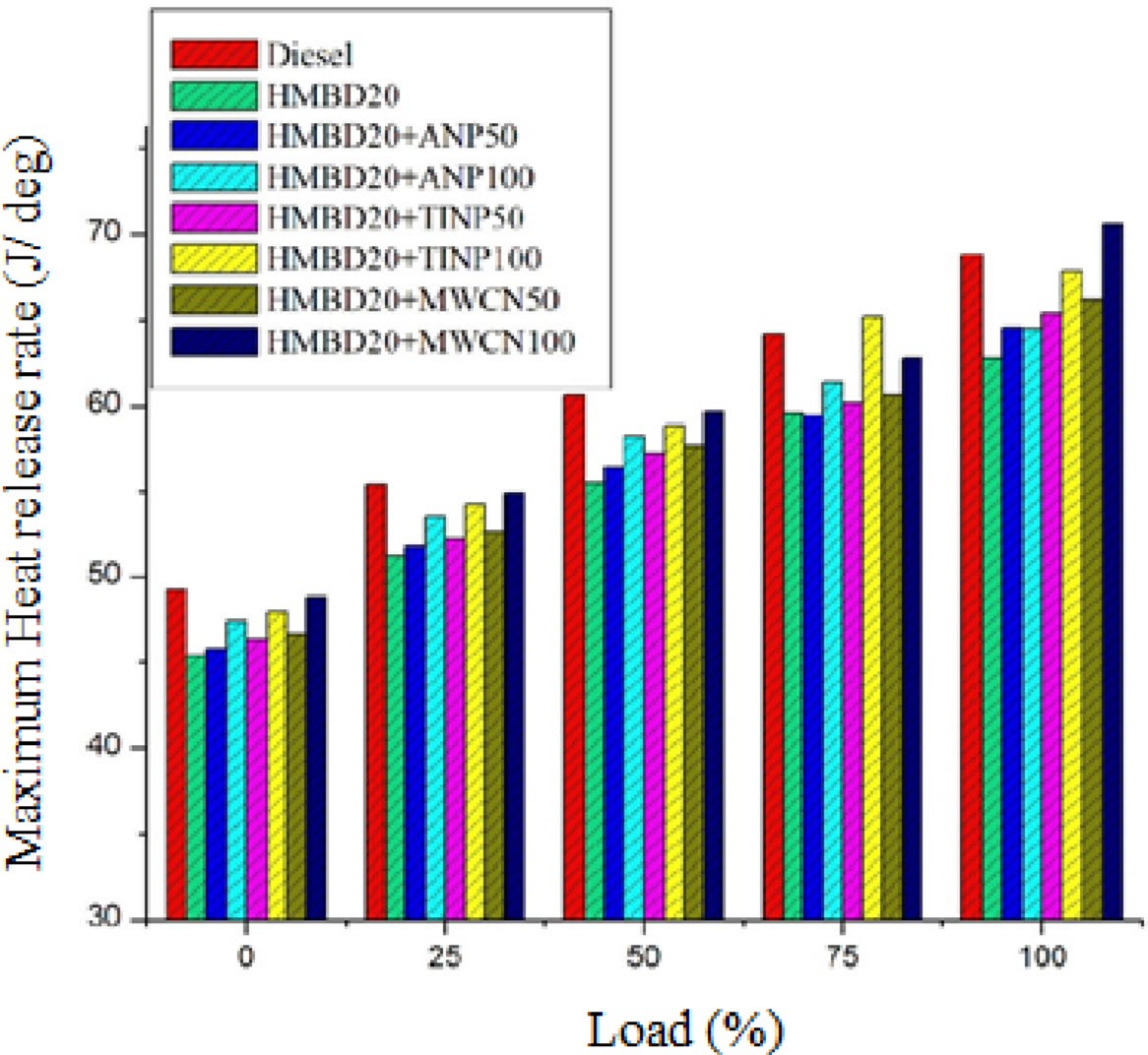
Variation of maximum heat release rate with load for all test fuels.

Figure 12 depicts the maximum HRR with engine load for all fuels. The maximum heat release rate signifies the energy release rate during combustion process after premixed stage. The diesel fuel exhibits highest HRR (69.6 J/^o^CA) compared to HMBD20 fuel (64.2 J/^0^CA) at peak load due to higher viscosity and lower calorific value of biodiesel. The addition of metal oxide NP’s (Alumina and Titania) to the biodiesel blend improves the HRR by a maximum of 3.12% and 6.28% than HMBD20 fuel due to enhanced fuel-air mixing and catalytic behavior of NPs^23^. The HMBD20 + MWCN50 and HMBD20 + MWCN100 fuel causes higher HRR by 4.68% and 8.96% than HMBD20 fuel at peak load. MWCNT’s possess higher heat transfer carrying capacity and reduced ignition delay causes higher heat release rate at both 50 ppm and 100 ppm concentration than HMBD20 fuel. Among all nano additives, MWCNT’s shows the most substantial enhancement of Maximum HRR due to intensified combustion process and catalytic behavior. The higher HRR obtained with NP’s enhanced fuels indicates a faster and more efficient combustion which contributes to improved BTE due to enhanced premixed combustion leads to better combustion phasing and more effective utilization of the fuel energy.

### CO emissions

**Fig. 13:**
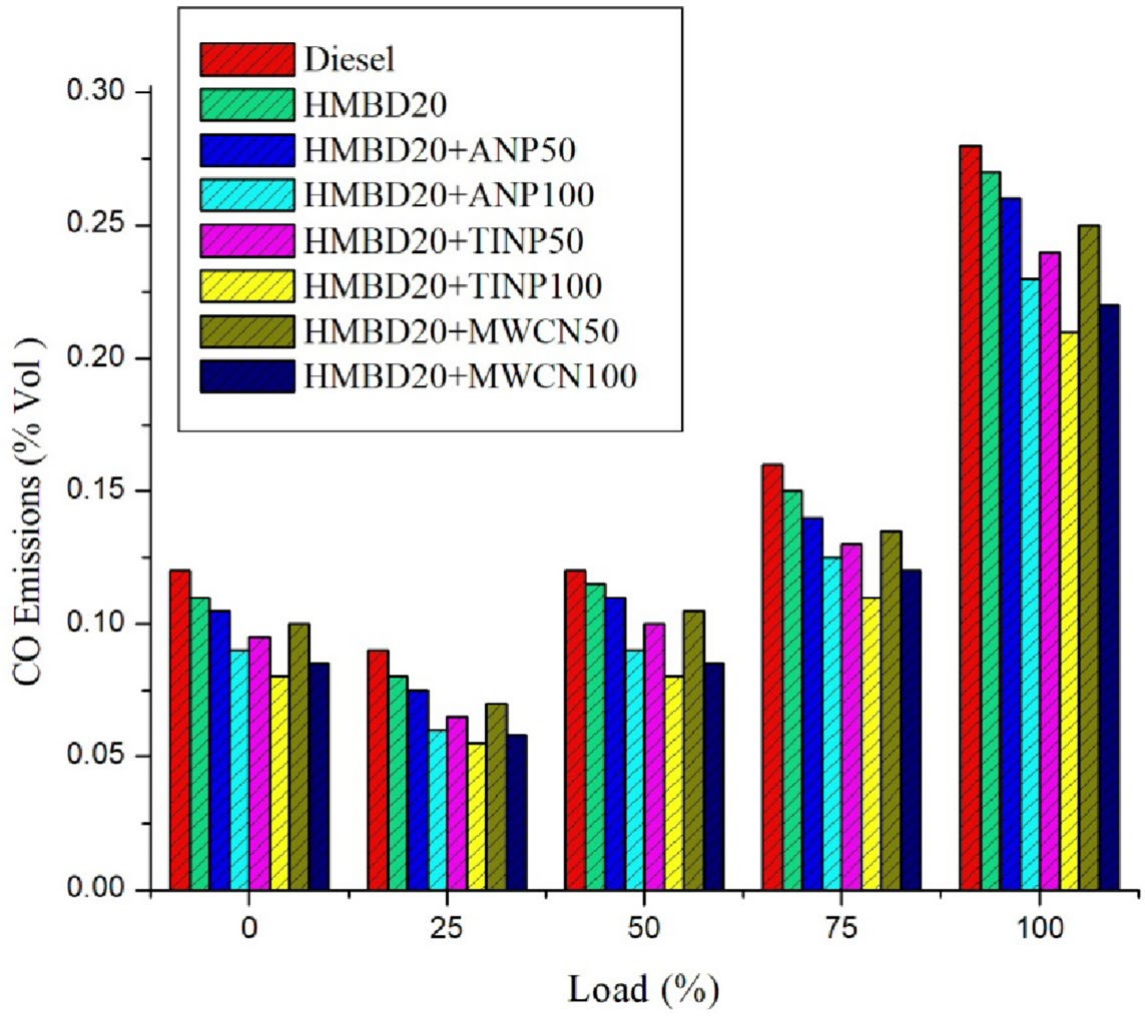
Variation of CO emissions with load for all test fuels.

Figure 13 depicts the CO emission values at varying loads for test fuels. At zero load condition, the CO emissions were highest for a partial load (25–50%) and consistent reductions in CO emissions were noted at high loads (50–100%). This behavior is attributed to poor fuel-air mixing, lower in-cylinder temperature and incomplete oxidation at low loads, whereas at higher loads provides the elevated combustion temperature and improved oxidation of CO to CO_2_. The CO emissions were slightly reduced by (3.26–8.56%) for HMBD20 fuel than diesel at all loads. A marginal reduction of CO pollutants for the HMBD20 + ANP50, HMBD20 + TINP50 and HMBD20 + MWCN50 fuels by 3.84%, 11.04%, 7.40% at peak load compared to HMBD20 fuel. This is due to higher surface-volume area of NP’s, increased turbulence, and better fuel-air mixing^24^. An increase in NP’s concentration to 100 ppm, CO pollutants was diminished by 14.81, 22.24 and 18.51% for the HMBD20 + ANP100, HMBD20 + TINP100 and HMBD20 + MWCN100 fuels, respectively. Improved atomization, better mixing of fuel-air mixture and limited oxygen availability effects the formation of CO pollutants^33^. The lower CO emissions also reduce the need for complex after-treatment systems making the nano enhanced biodiesel a viable and environmentally friendly fuel for stationary power generation applications.

### CO_2_ emissions

**Fig. 14:**
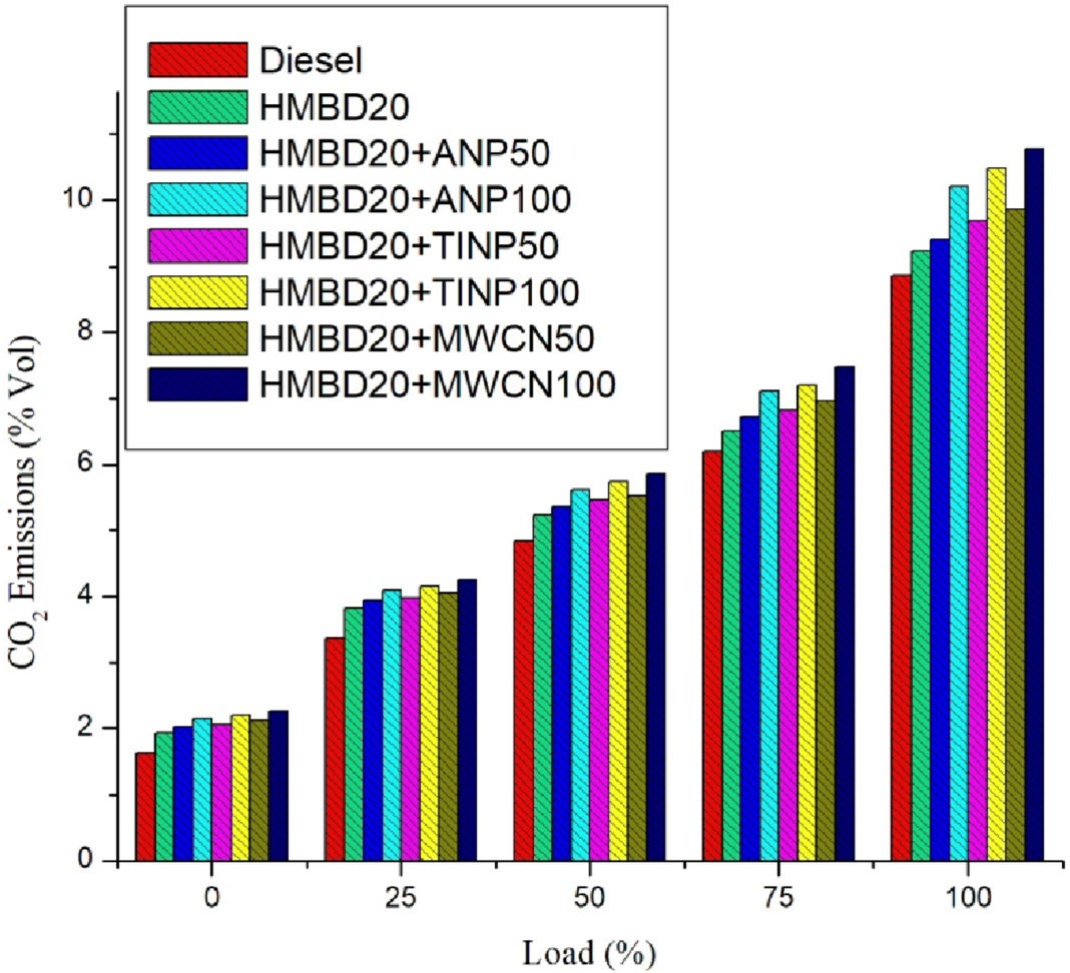
Variations of CO_2_ emissions with load for all test fuels.

Figure 14 depicts the CO_2_ emission values at varying loads for test fuels. CO2 emissions were rising with increase in load due to higher fuel flow and more oxidation at elevated temperatures. The oxygenated nature of HMBD20 fuel produces 4.17% higher CO_2_ emissions than HMBD20 fuel at peak load, primarily because of its inherent oxygen content, which promotes complete combustion and facilities the conversion of CO_2_ emissions. The inclusion of ANP, TINP and MWCNT’s at 50 ppm and 100 ppm level enhances the CO_2_ emissions by 1.94%, 4.76%, 6.70%, 10.49%, 13.41% and 16.66% respectively at peak load. This is due to catalytic activity of NP’s which promotes better atomization, improved fuel-air mixing accelerates the oxidation with carbon elements. Shorter ignition delay and superior oxygen content at higher loads favor the complete conversion of CO to CO_2_ emissions^45^. The increase in CO_2_ emissions indicates more complete combustion and reduced formation of incomplete combustion products such as CO and HC, which is desirable for achieving cleaner engine operation^58^.

### HC emissions

**Fig. 15:**
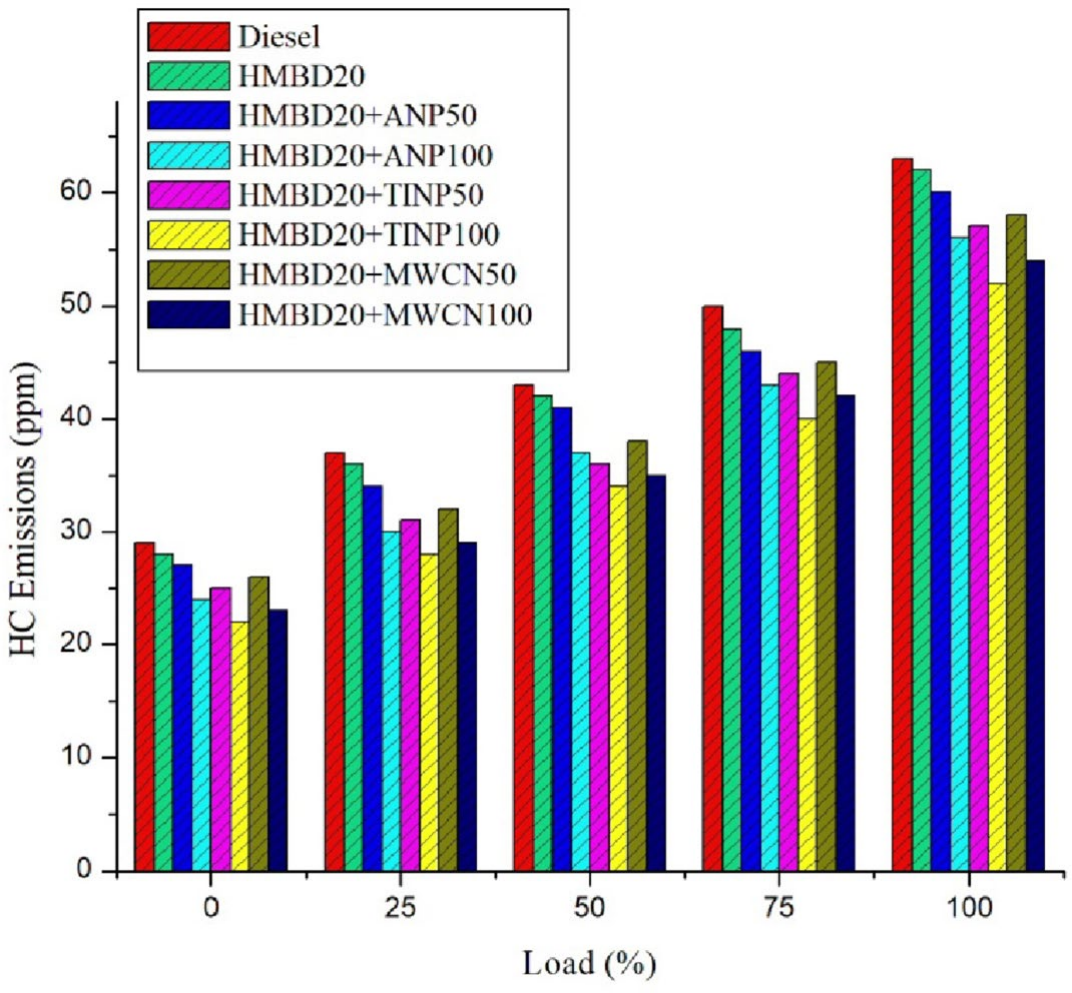
Variation of HC emissions with load for all test fuels.

Figure 15 shows the variations of HC emissions with load for test fuels. The HC emissions were reduced by (1.58%−3.64%) for HMBD20 fuel than diesel due to oxygenated nature of biodiesel, which promotes complete oxidation of fuel and reduces the formation of fuel-rich zones. The enhanced catalytic action and improved thermal conductivity of NP’s promote the oxidation of unburned hydrocarbons causes a reduction of HC emissions by 3.22%, 8.06% and 6.45% for the HMBD20 + ANP50, HMBD20 + TINP50 and HMBD20 + MWCN50 fuels at peak load^25^. Improved flame propagation and homogeneous combustion environment aided by the NP’s, enhances the combustion rate causes reduction of the HC emissions by 9.67, 16.12 and 12.90% for the HMBD20 + ANP100, HMBD20 + TINP100 and HMBD20 + MWCN100 fuels at peak load^34^. The lowest HC emissions were obtained for the HMBD20 + TINP100 fuel by 16.12% and 17.46% compared to HMBD20 and diesel fuel at peak load. This behavior is attributed to enhanced catalytic activity, high thermal conductivity and larger surface area accelerates the evaporation and intensifies the flame propagation thereby creating a more homogeneous combustion environment which meets the stringent emission norms^58^.

### NOx emissions

**Fig. 16:**
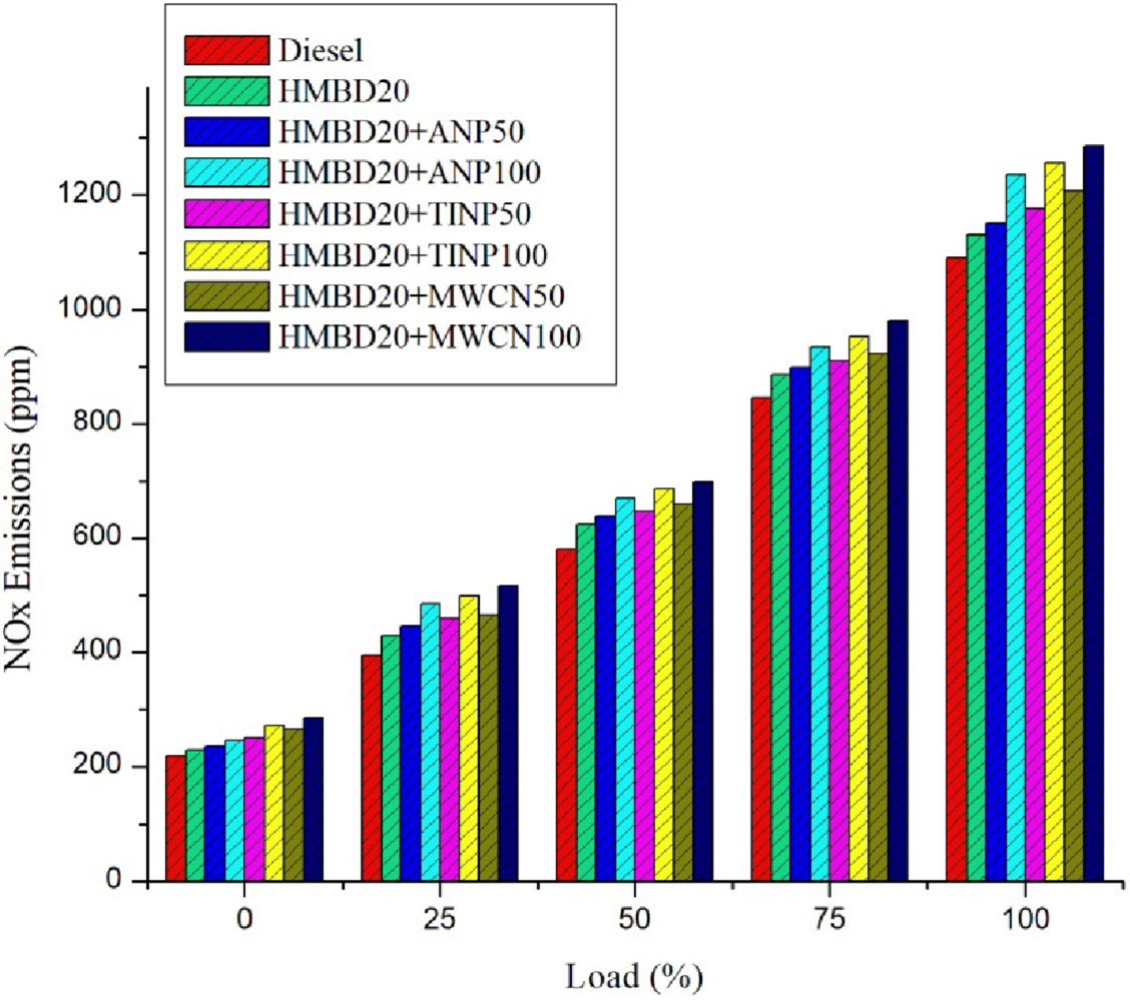
Variation of NOx emissions with load for all test fuels.

Figure 16 shows the variations of NOx emissions with load for test fuels. The NOx emissions for HMBD20 fuel were raised by 3.66% than diesel due to rich oxygen content of biodiesel and the resulting increase in local combustion temperature and availability of excess oxygen for NO_x_ formation. With the inclusion of NP’s in HMBD20 fuel, the NOx emissions were enhanced by 1.73, 4.07, 6.63% for the HMBD20 + ANP50, HMBD20 + TINP50 and HMBD20 + MWCN50 fuels at peak load compared to HMBD20 fuel due to elevated combustion temperatures in the engine cylinder^26^. Higher NOx emissions were noted for the HMBD20 + ANP100, HMBD20 + TINP100 and HMBD20 + MWCN100 fuels compared to HMBD20 fuel by 9.29, 11.15 and 13.71% at full load because of enhanced heat transfer and intensified oxidation process leads to NOx pollutants formation^27,35^ The high thermal conductivity and catalytic activity of the NP’s intensifies the combustion process, leading to higher peak temperature and longer residence time of high temperature gases favors the NOx formation.

### Smoke opacity

**Fig. 17:**
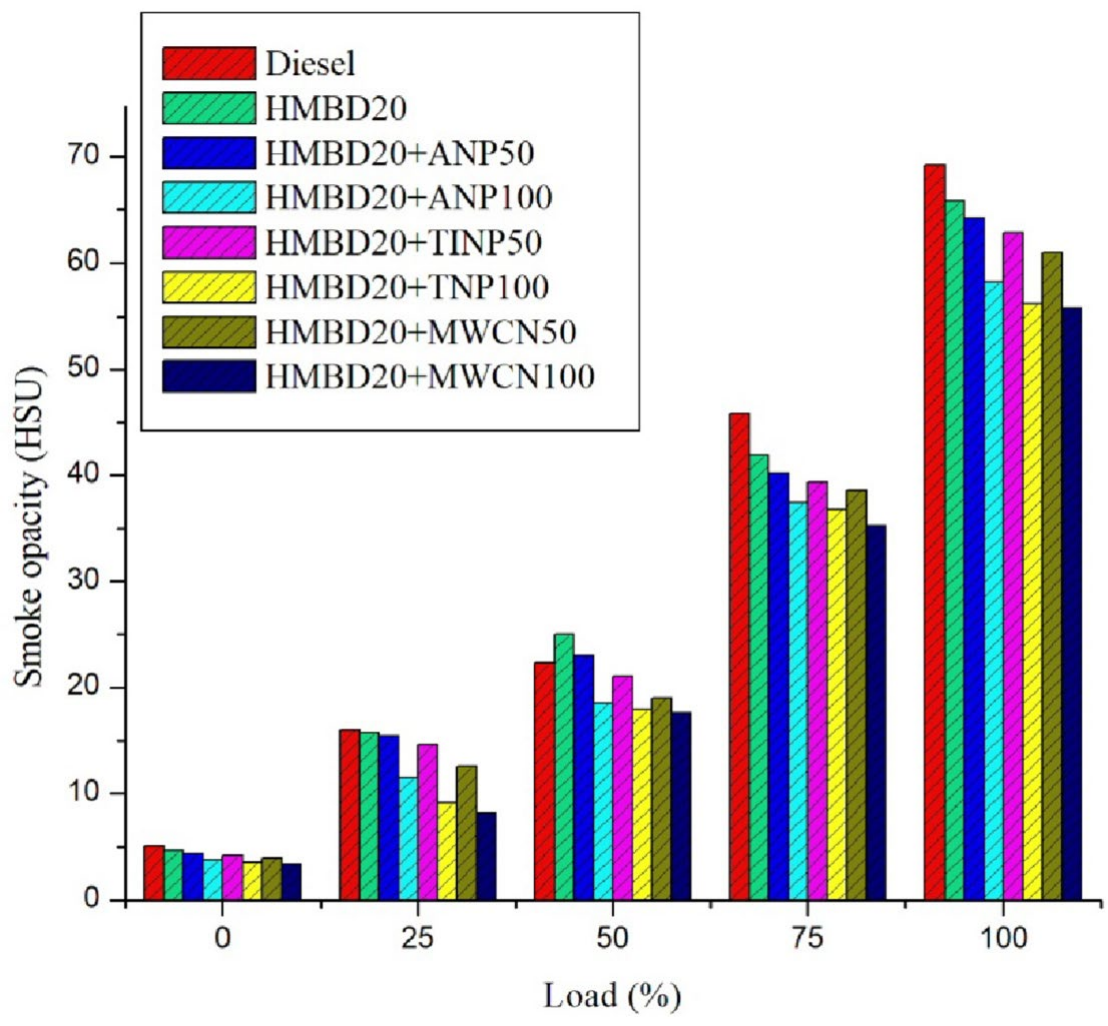
Variation of smoke emissions with load for all test fuels.

Figure 17 depicts the variation of Smoke emissions with load for test fuels. The smoke emissions for HMBD20 fuel reduce by 4.91% than diesel fuel at peak load, majorly due to the inherent oxygen content of biodiesel, promotes the oxidation of soot particles and reduces the formation of fuel-rich diffusion zones. The addition of NP’s (TiO_2_, Al_2_O_3_) and MWCNT’s into the HMBD20 fuel reduces the smoke emissions by 2.43, 4.55 and 7.44% with the 50-ppm concentration and further reduced by 11.55, 14.58 and 15.19% with the 100-ppm concentration at peak load. The rapid evaporation of fuel droplets and improved air-fuel mixing of the nano additive fuels leads to enhancement in the combustion of fuel^28^. The HMBD20 + TINP100 fuel shows lowest smoke emissions compared to all remaining fuels because TiO_2_ NPs are more effective due to its oxygen buffering ability, better dispersion stability^29^. The factors such as improved atomization, rapid evaporation of fuel droplets and enhanced fuel-air mixing caused by the micro-explosion phenomenon and the high surface area to volume ratio of nanoparticles additive fuel samples promote the oxidation of soot particles during the diffusion combustion phase.

## Machine learning approach

In this section, Machine learning models such as Decision Tree (DT), Support Vector Machine (SVM) and Artificial Neural Network (ANN) were chosen to predict the engine parameters using experimental data. The prediction accuracy of these models was evaluated by using performance metrics such as R^2^, MSE, MAE and RMSE. These metrics were useful to establish best suitable fuel sample and most efficient ML model for prediction of engine behavior.

### BTE

The performance of the DT, SVM and ANN (MLP) model to predict BTE was evaluated using performance metrics. The Table 5 shows the Performance metrics of BTE and BSFC. The DT model achieved a high R^2^ of 0.9899 and 0.9088 for training and testing data reveals a strong predictive capability with less overfitting of the model. The MAE and RMSE values are 0.418 & 1.085, 0.56 & 1.25 for training and testing data, respectively. The SVM model obtained R^2^ of 0.9571, 0.9620 and 0.9592 for training, testing and total data which shows the consistency of the model. The MAE and RMSE values are 0.773, 0.817 & 0.782; 1.064, 0.994 & 1.049 for training, testing, and overall data, respectively. The ANN model shows R^2^ of 0.8572, 0.0.6055 and 0.8048 for training, testing and total data shows the under fitting of the data and drop in effectiveness of the model. The MAE and RMSE values are 1.437, 2.164 & 1.664; 1.867, 3.028 & 2.294 for training, testing, and overall data, respectively. The DT model exhibits the most efficient and robust model for predicting of BTE of an engine^46^.

**Table 5:** Performance metrics of BTE & BSFC.

Performance metrics		BTE			BSFC		
		DT	SVM	ANN	DT	SVM	ANN
R^2^	Training data	0.9899	0.9571	0.8571	0.998	0.9706	0.9100
	Testing data	0.9088	0.9620	0.6054	0.7710	0.8773	0.8863
	Total data	0.9738	0.9591	0.8047	0.9661	0.9463	0.9122
MAE	Training data	0.4187	0.7726	1.4366	0.0000	0.0188	0.0270
	Testing data	1.0854	0.8172	2.1637	0.0355	0.0346	0.0326
	Total data	0.6265	0.7823	1.6638	0.0111	0.0237	0.0288
MSE	Training data	0.3141	1.1317	3.4861	0.0000	0.0004	0.0011
	Testing data	1.5653	0.9884	9.1681	0.0016	0.0016	0.0015
	Total data	0.7051	1.1004	5.2617	0.0005	0.0008	0.0012
RMSE	Training data	0.5604	1.0638	1.8671	0.0000	0.0193	0.0336
	Testing data	1.2511	0.9942	3.0278	0.0394	0.0406	0.0390
	Total data	0.8397	1.0490	2.2938	0.0220	0.0277	0.0354

**Fig. 18:**
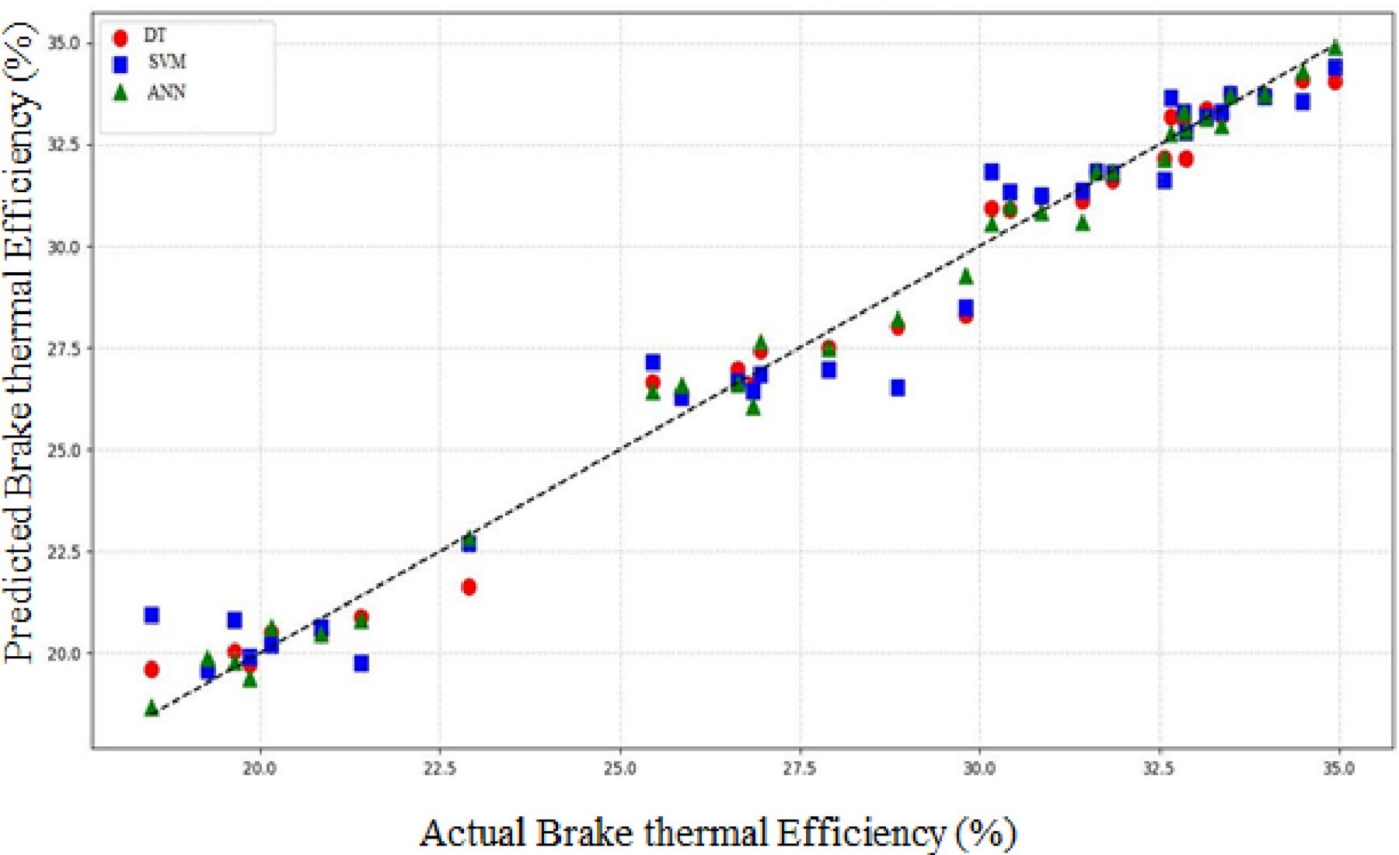
Predicted versus actual BTE values of all models.

Figure 18 illustrates the close agreement between predicted and actual BTE values for all models. The DT, SVM and ANN models were accurately predicted BTE across various fuel samples at different loads with minimal error. The DT model shows a BTE of 34.8% for HMBD20 + MWCN100 and yielding a predicted value of 33.59% with an error of 2.6%. The SVM model exhibits exceptionally low prediction errors for all fuels and accurate predicted values were noted for HMBD20 + MWCN100 and HMBD20 + TINP100 of 33.50% and 33.60% at peak BP of 4.94 kW. In the ANN model, the predicted values were closely matched with experimental values and especially for the HMBD20 + MWCN100 fuel predicted a BTE value of 34.25% at peak BP (4.94 kW)^20^. It shows the consistency of the model and especially for the nano additive fuels in case of BTE prediction^37^. Overall, ANN was outperformed in BTE prediction with highest accuracy than SVM and DT models^47^.

### BSFC

The performance of the DT, SVM and ANN (MLP) model to predict BSFC was evaluated using performance metrics. The DT model for BSFC prediction shows excellent performance for training data set (R^2^ = 0.998) and overall data (R^2^ = 0.9661) meanwhile reduce its accuracy (R^2^ = 0.7710) for testing data set due to overfitting. The MAE and RMSE for testing, training and overall data set were noted as 0.01020, 0.0355, 0.0111 and 0.0264, 0.0394, 0.0220, respectively. The SVM model for BSFC prediction shows a strong generalization with R^2^ of 0.9706, 0.8773 and 0.9463 for training, testing and overall data indicates the robust performance^38^. The ANN model shows a consistent performance in predicting R^2^ of 0.9100, 0.9122, and 0.8863 for training, testing and overall data confirms the stable accuracy across all data sets^48^.

**Fig. 19:**
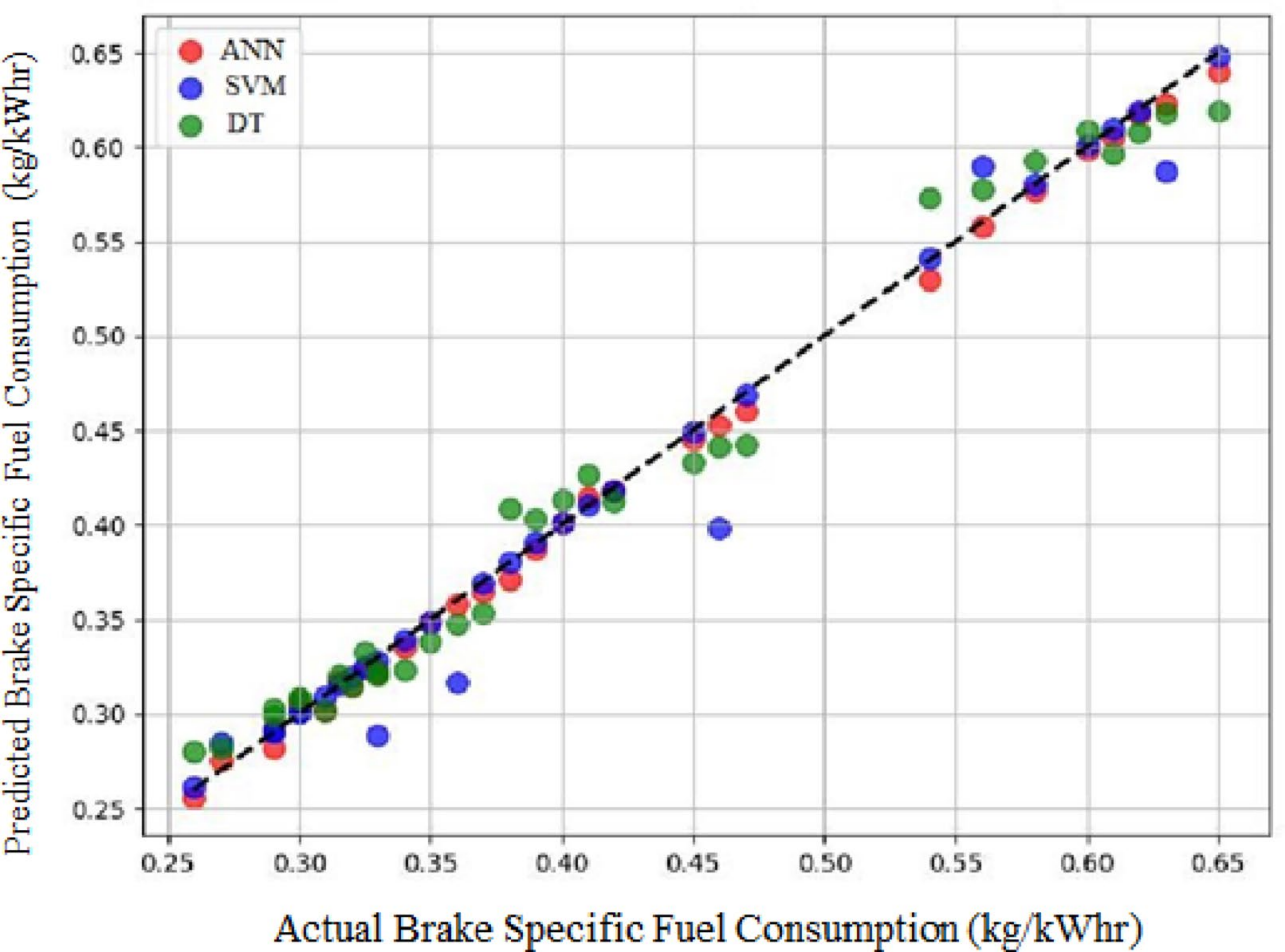
Predicted versus actual BSFC values of all models.

The predicted and actual BSFC values of all models were shown in Fig. 19. The comparison of actual and predicted values for the model reveals that predictions were more accurate at low and medium level loads (1.30–3.75 kW) for nano additive biodiesel blended fuels in the DT. A Minor deviation was observed for diesel and HMBD20 fuel at higher loads shows higher predictive capability. The SVM model demonstrates high degree of predictive accuracy with minimal deviations i.e., less than 0.03 kg/kWhr for NP’s blended fuels, especially at low loads and also shows more reliability in modeling. The ANN model clearly identified the trend analysis of decreasing BSFC with engine load and with NP’s enriched blends. Finally, In the prediction of BSFC, DT exhibits a remarkably close match with actual values, SVM shows strong generalization with more stability, but ANN requires more tuning to reduce the deviations between various conditions^49^.

### EGT

The performance of the DT, SVM and ANN (MLP) model to predict EGT was evaluated using performance metrics. Table 6 shows the Performance metrics of EGT, Peak cylinder pressure and Maximum HRR. The DT model exhibits a strong predictive capability for EGT with R^2^ of 0.93, 0.76 and 0.88 for training; testing and overall data which confirms the better generalization of the model. The MAE and RMSE values for the DT model are 15.11 °C, 31.16 °C, 19.93 °C and 19.82 °C, 36.77 °C, 26.09 °C respectively suggests that a slight overfitting of the model leads to an improvement of the model. The SVM model shows an improvement in performance metrics such as R^2^ of 0.9429, 0.8895, 0.9278 for training; testing and overall data indicates the model’s capability with minimal deviation. The ANN model yields a moderate accuracy in predicting EGT with R^2^ of 0.7773, 0.7479 and 0.7705 which requires learning capability while capturing the trend with minimal error margins.

**Table 6:** Performance metrics of EGT, peak cylinder pressure and Max. HRR.

Performance metrics		EGT			Peak Cyl. Pr			HRR		
		DT	SVM	ANN	DT	SVM	ANN	DT	SVM	ANN
R^2^	Training data	0.9323	0.9429	0.7773	0.9971	0.9921	0.9864	0.9968	0.9883	0.9957
	Testing data	0.7601	0.8895	0.7478	0.9725	0.9806	0.8737	0.9636	0.941	0.9658
	Total data	0.8826	0.9278	0.7705	0.9893	0.9885	0.951	0.986	0.9794	0.99
MAE	Training data	15.119	11.7507	29.5434	0.2857	0.5378	0.771	0.2857	0.6187	0.5816
	Testing data	31.166	23.6506	31.4501	1.2917	1.133	2.733	1.8889	2.3256	1.8504
	Total data	19.9333	15.3207	30.1154	0.5875	0.7164	1.3597	0.7667	0.96	0.8353
MSE	Training data	393.095	331.624	1293.45	0.2143	0.5728	0.9891	0.3571	1.4584	0.5398
	Testing data	1352.74	623.03	1421.7	2.1458	1.5112	9.841	4.5185	6.8062	3.948
	Total data	680.988	419.04	1331.92	0.7937	0.8543	3.6446	1.6056	2.528	1.2215
RMSE	Training data	9.8266	18.2106	35.9646	0.4629	0.7568	0.9945	0.5976	1.2076	0.7347
	Testing data	36.7796	24.9607	37.7054	1.4649	1.2293	3.137	2.1257	2.6089	1.987
	Total data	26.0957	20.4707	36.4956	0.8909	0.9243	1.9091	1.2671	1.59	1.1052

**Fig. 20:**
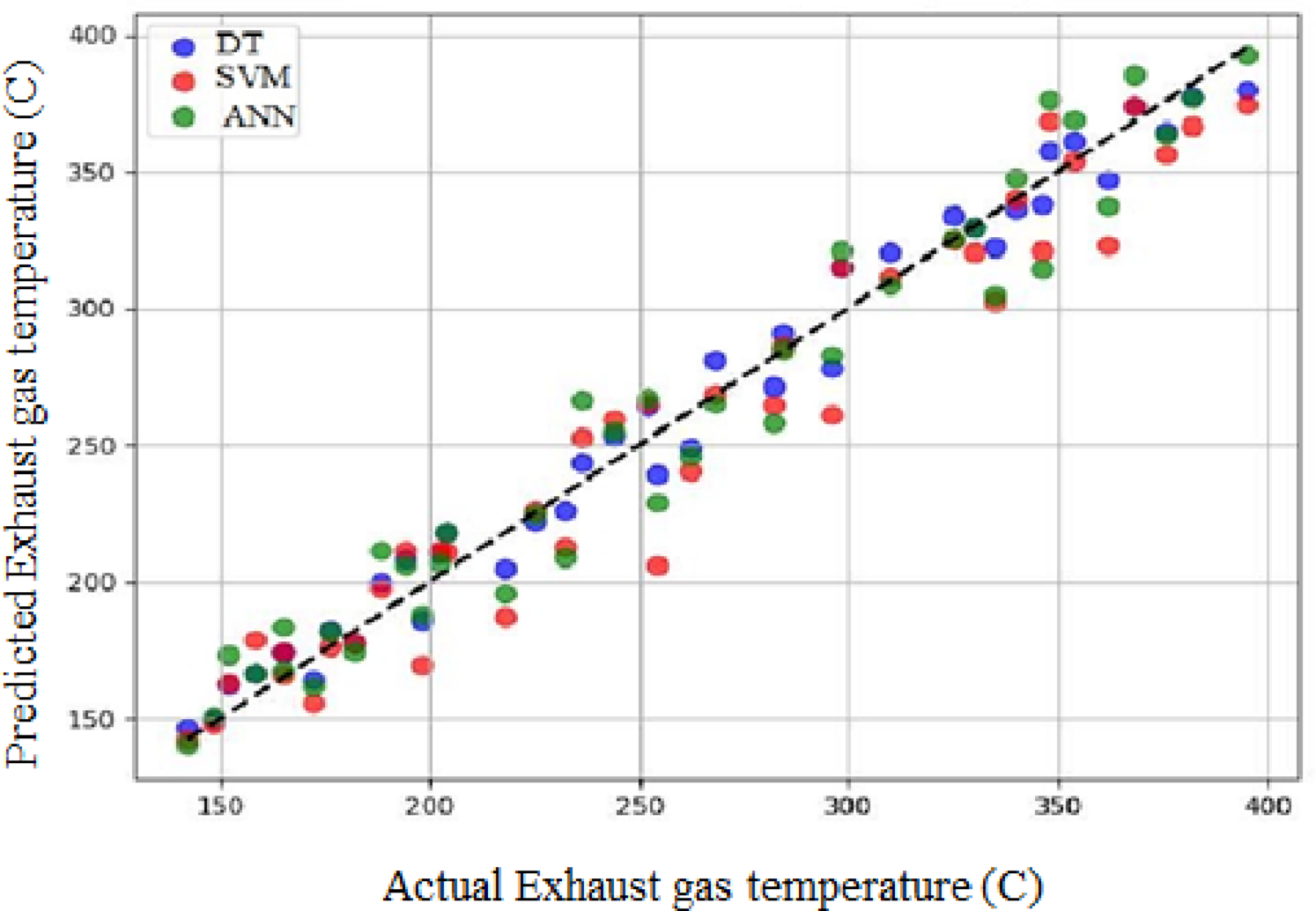
Predicted versus actual EGT values of all models.

The predicted and actual EGT values of all models were represented in Fig. 20. The prediction of EGT from the figure exhibits a strong alignment with actual data at lower and medium loads (0–50%) and at high loads (75–100%) it will be low especially for nano additive biodiesel fuels. In this SVM model, lowest absolute error (0.099 °C) was found for the HMBD20 + TINP50 fuel at 75% load. The ANN model maintains better accuracy at medium loads, the HMBD20 + ANP50 at zero load shows an absolute error of 0.32 °C. Among three models, SVM exhibits the lowest error and highest accuracy in predicting EGT while ANN was highly accurate at low loads and DT shows greater error margins^50^.

### Peak cylinder pressure (PCP)

The performance of the DT, SVM and ANN (MLP) model to predict peak cylinder pressure was evaluated using performance metrics. The DT model shows superior accuracy, achieving R^2^ of 0.9971, 0.9725 and 0.9893, lowest MAE, RMSE of 0.2857, 1.2917 and 0.5875; 0.4629, 1.4649 and 0.8909 for training, testing, and overall data, respectively. The SVM model was outperformed with R^2^ of 0.9921, 0.9806 and 0.9885, moderate MAE, RMSE of 0.5378, 1.1330 and 0.7164; 0.7568, 1.2293 and 0.9243 for training, testing, and overall data, respectively. The ANN model shows reasonable accuracy achieving R^2^ of 0.9864, 0.8737 and 0.9510, lowest MAE, RMSE of 0.7710, 2.733 and 1.3597; 0.9945, 3.1370 and 1.9091 for training, testing, and overall data, respectively. Among all SVM models more dependable and DT model were most effective while predicting peak cylinder pressure.

**Fig. 21:**
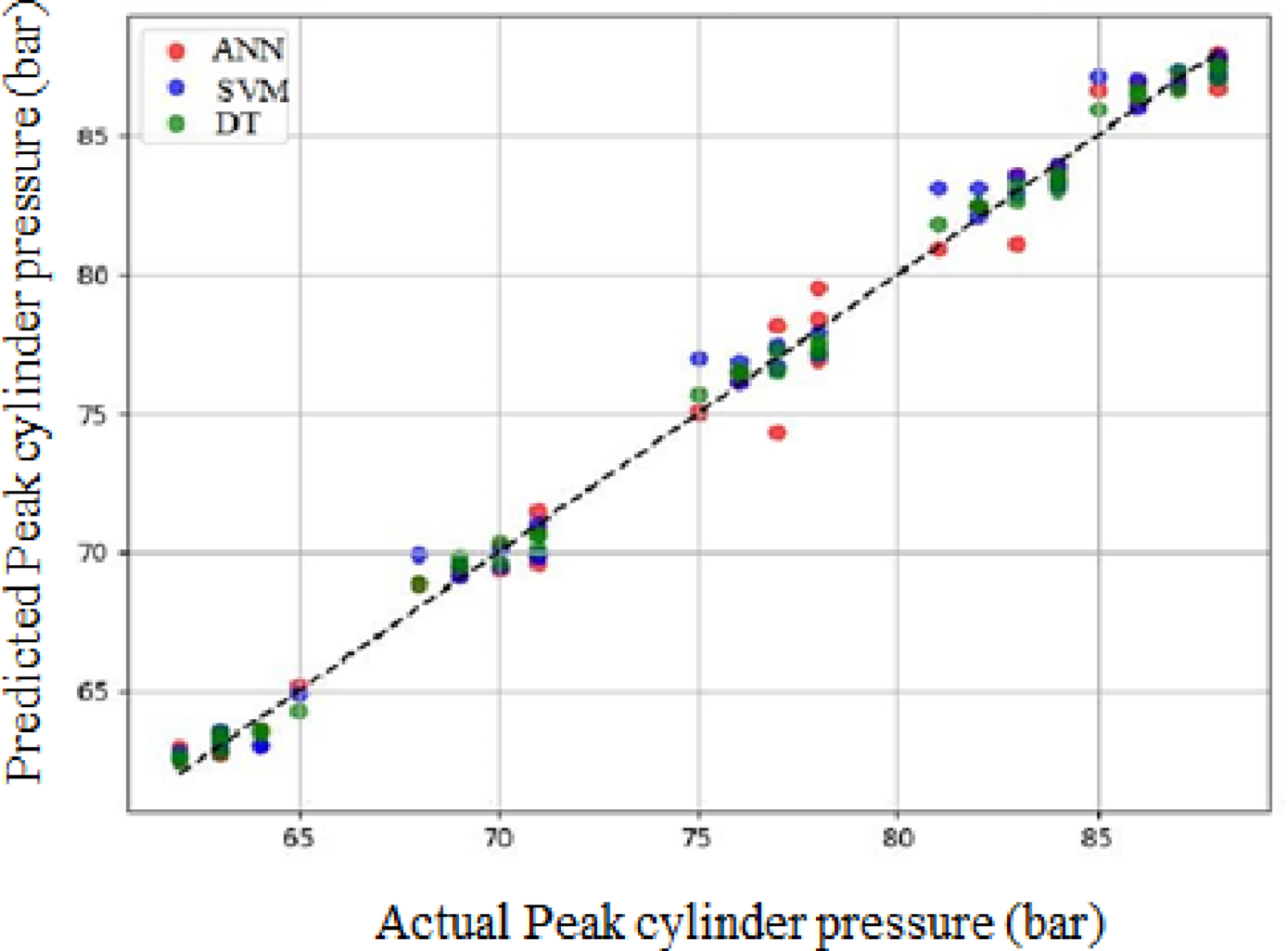
Predicted versus actual peak cylinder pressure values of all models.

The predicted versus actual PCP values for all models was shown in Fig. 21. The DT model accurately predicted peak cylinder pressure for all fuels and various loads with lowest error. The HMBD20 + MWCN50 fuel at full load actual value shows very identical with predicted value which confirms its accuracy. The SVM model exhibits more deviation at peak load especially for nano additive fuels meanwhile it shows a strong predictive capability for remaining fuels and loads. The ANN model also captures good relations for nano additive fuels with effective model generalization ability. Overall, DT model shows a higher accuracy at all loads, but SVM model performs better at low and medium loads^51^.

### Maximum HRR

The performance of the DT, SVM and ANN (MLP) model to predict Maximum HRR was evaluated using performance metrics. The DT model shows best accuracy with R^2^ of 0.9968, MAE of 0.2857 and RMSE of 0.5976 during training, R^2^ of 0.9636, MAE of 1.8889 and RMSE of 2.1257 during testing and R^2^ of 0.9860, MAE of 0.7667 and RMSE of 1.2671 for overall data sets. The SVM model shows an R^2^ of 0.9883, MAE of 0.6187 and RMSE of 1.2076 during training, R^2^ of 0.9410, MAE of 2.3256 and RMSE of 2.6089 during testing and R^2^ of 0.9794, MAE of 0.9600 and RMSE of 1.590 for overall data sets. In overall, DT offers superior accuracy and minimal error, SVM exhibits highest variance using all data sets.

**Fig. 22:**
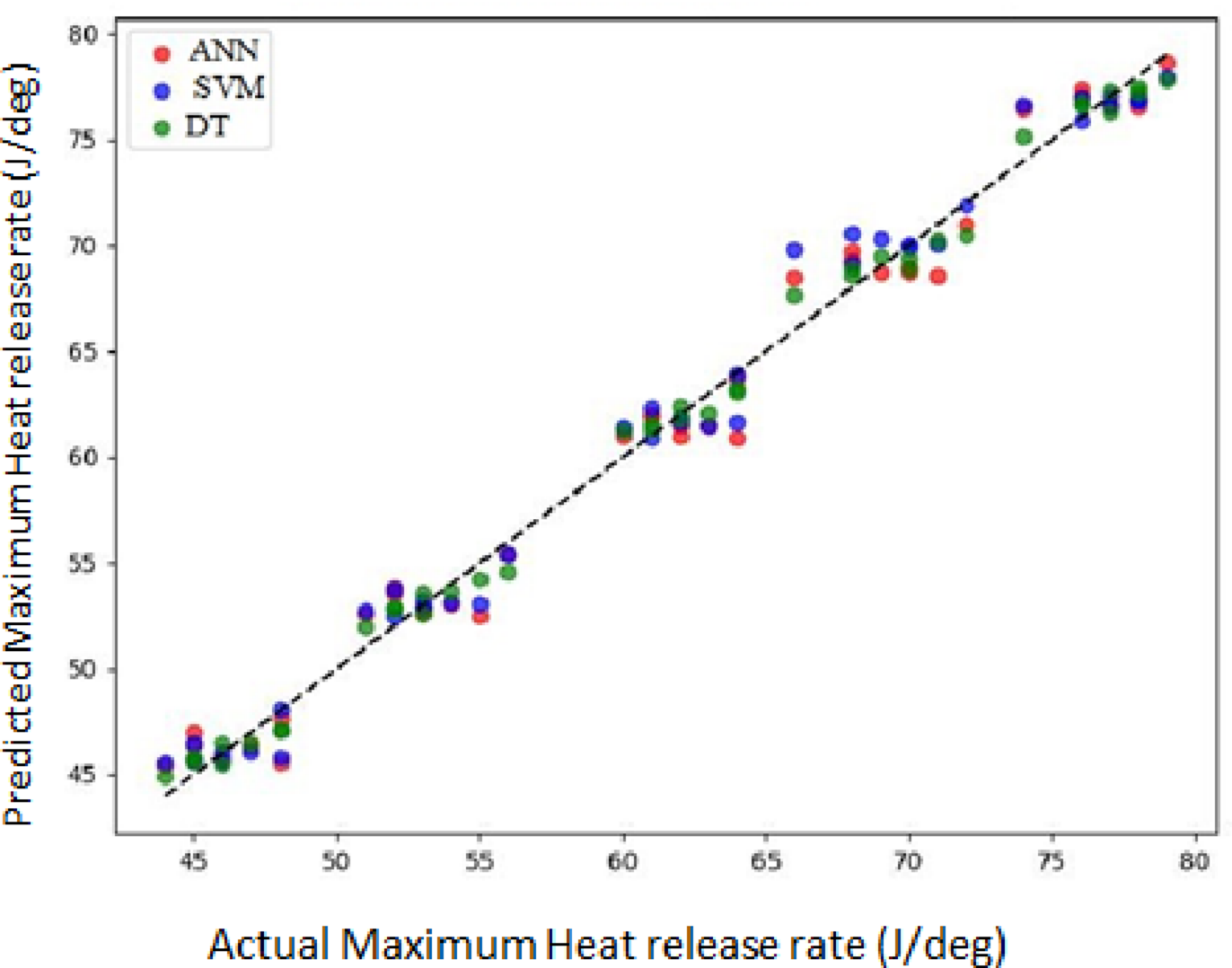
Predicted versus actual of maximum heat release rate values of all models.

The predicted versus actual values of all models for the Max HRR was shown in Fig. 22. The DT model predicts accurately the nano additive fuels at peak loads showing an error of less than % with actual values thus indicates the effectiveness of capturing trends with experimental values. The SVM model also predicts efficiently, showing the minimal errors between predicted and actual values, especially at peak loads. The ANN model shows an excellent consistency in predicting HRR for all fuels at all loads. This model exhibits minimal deviation errors among predicted and actual values across all fuels. Among all models, ANN exhibits greater prediction accuracy with more consistency^52^.

### CO emissions

The performance of the DT, SVM and ANN (MLP) model to predict CO emissions was evaluated using performance metrics. Table 7 shows the Performance metrics of CO, CO_2,_ and HC emissions. The DT model was outperformed with highest R^2^ of 0.9865, 0.9424 and 0.9641, lowest MAE and RMSE of 0.052. 0.0190, 0.0093 and 0.0061, 0.0198, 0.0120 for training, testing, and overall data sets. The SVM model shows good R^2^ of 0.9736, 0.8297 and 0.9004; MAE and RMSE of 0.0072. 0.0255, 0.0127 & 0.0086, 0.0341, 0.0200 for training, testing, and overall data sets. The ANN model performs well R^2^ of 0.9724, 0.9313 and 0.9516; MAE and RMSE of 0.0071, 0.0194, 0.0108 and 0.0087, 0.0216, 0.0139 for training, testing, and overall data sets. Among all models, DT emerged as the most dependable model followed by ANN, while SVM exhibits lowest performance for predicting CO emission^53^.

**Table 7:** Performance metrics of CO, CO2, and HC emissions.

Performance metrics		CO			CO_2_			HC		
		DT	SVM	ANN	DT	SVM	ANN	DT	SVM	ANN
R^2^	Training data	0.9865	0.9736	0.9724	0.9989	0.9898	0.9897	0.9899	0.9761	0.9472
	Testing data	0.9424	0.8297	0.9313	0.9774	0.9647	0.9609	0.9375	0.8999	0.9516
	Total data	0.9641	0.9004	0.9516	0.996	0.9856	0.9848	0.9664	0.942	0.9493
MAE	Training data	0.0052	0.0072	0.0071	0.0137	0.1597	0.2119	0.6429	1.1238	1.7525
	Testing data	0.019	0.0255	0.0194	0.3068	0.3457	0.4255	3.2917	3.8702	2.6885
	Total data	0.0093	0.0127	0.0108	0.072	0.1969	0.2546	1.4375	1.9477	2.0333
MSE	Training data	0.000037	0.000073	0.000077	0.0011	0.074	0.0753	1.0476	2.4713	5.467
	Testing data	0.000392	0.00116	0.000468	0.135	0.211	0.2346	12.3125	19.7302	9.534
	Total data	0.000144	0.000399	0.000194	0.028	0.1019	0.1071	4.4271	7.649	6.6871
RMSE	Training data	0.0061	0.0086	0.0087	0.033	0.2731	0.2744	1.0235	1.572	2.3382
	Testing data	0.0198	0.0341	0.0216	0.3683	0.4598	0.4843	3.5089	4.4419	3.0877
	Total data	0.012	0.02	0.0139	0.1674	0.3193	0.3274	2.1041	2.7657	2.5859

**Fig. 23:**
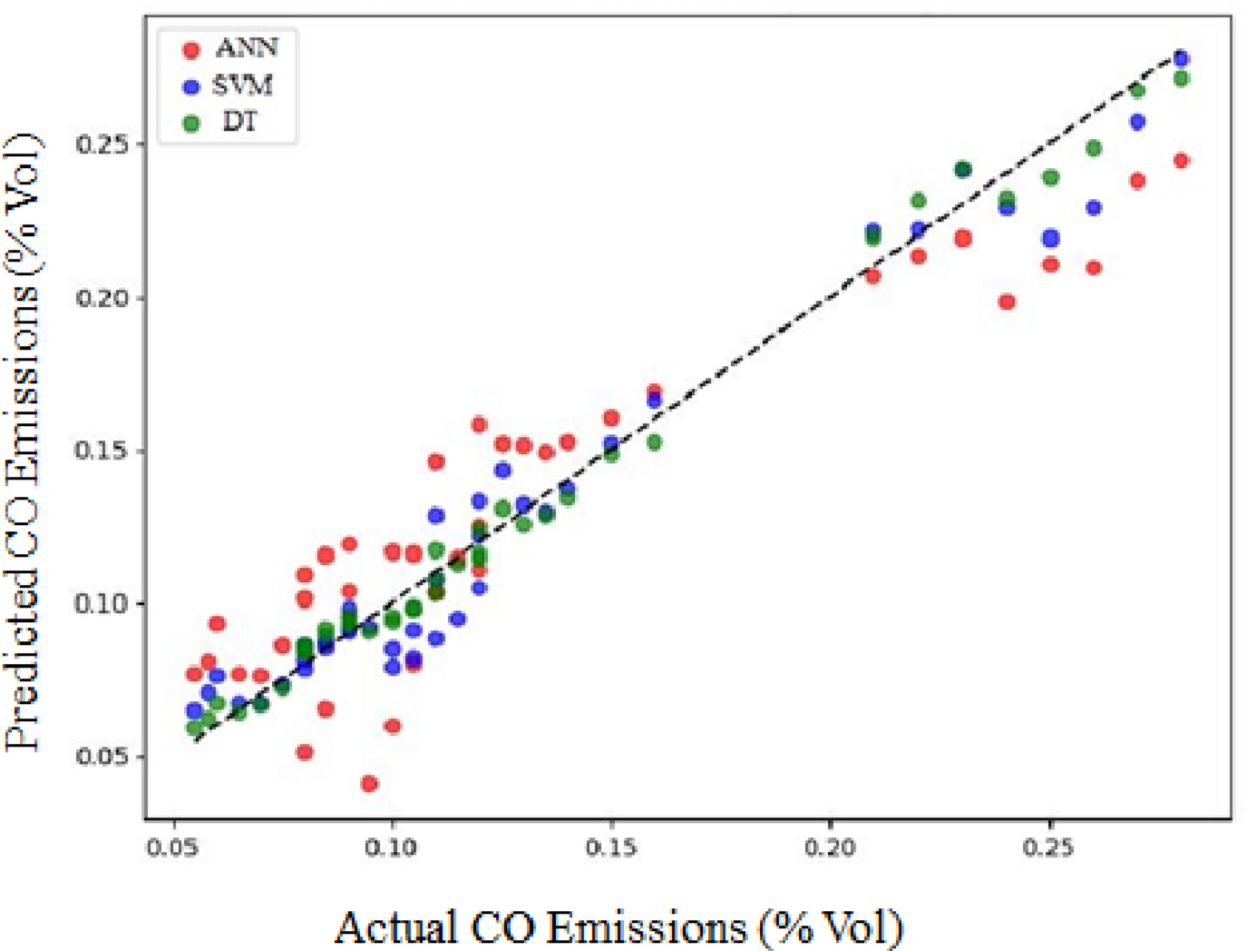
Predicted versus actual CO emissions of all models.

The predicted versus actual CO values of all models were shown in Fig. 23. The DT model exhibits a strong relation between predicted and actual CO emissions at all loads. At peak loads, the HMBD20 + MWCN100 fuel predicts CO emissions (0.2489%) nearer to the actual value (0.220%) shows ability of model while capturing the trends using nano additive biodiesel blended fuels. The SVM model predicts CO emissions with reasonable accuracy, especially at low to mid (0–50%) loads and for nano particle blends shows consistent trends like actual values affirms the model reliability^39^. The ANN model represents high consistency in predicting CO emissions for all fuels. At 25% load, HMBD20 + MWCN50 fuel shows the actual CO of 0.070 while predicted value of 0.0704 confirms lowest deviation among all fuels^44^.

### CO_2_ emissions

The performance of the DT, SVM and ANN (MLP) model to predict CO_2_ emissions were evaluated using performance metrics. The DT model was outperformed with highest R^2^ of 0.9989, 0.9774 and 0.9960, lowest MAE and RMSE of 0.0137. 0.3068, 0.072 and 0.033, 0.3683, 0.1674 for training, testing, and overall data sets. The SVM model shows good R^2^ of 0.9898, 0.9647 and 0.9856; MAE and RMSE of 0.1597, 0.3457, 0.1969 & 0.2731, 0.4598, 0.3193 for training, testing, and overall data sets. The ANN model performs well R^2^ of 0.9897, 0.9609 and 0.9848; MAE and RMSE of 0.2119, 0.4255, 0.2546 and 0.2744, 0.4843, 0.3274 for training, testing, and overall data sets. Among all models, ANN provides best balance of accuracy and generalization, but DT model is useful for quick and most accurate prediction capability^36^.

**Fig. 24:**
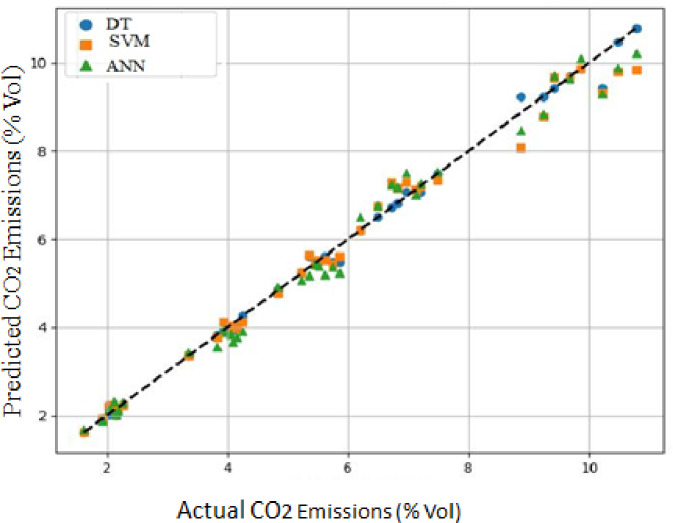
Predicted versus actual CO_2_ emissions of all models.

The predicted versus actual CO_2_ values of all models were shown in Fig. 24. All three models exhibited a strong correlation with the experimental values indicated by high determination coefficients. The DT model achieved the highest accuracy and ability to fit the experimental data with minimal scattering, especially at low and medium loads. The SVM model demonstrates stable prediction ability with minor deviations at medium level loads. The ANN model revealed a high prediction accuracy and well aligned with the reference line in capturing the nonlinear behavior of the data^54^.

### HC emissions

The performance of the DT, SVM and ANN (MLP) model to predict HC emissions was evaluated using performance metrics. The DT model was outperformed with highest R^2^ of 0.9899, 0.9375 and 0.9664, lowest MAE and RMSE of 0.6429, 3.2917, 1.4375 and 1.0235, 3.5089, 2.1041 for training, testing, and overall data sets. The SVM model exhibits a better R^2^ of 0.9761, 0.8999 and 0.9420, MAE and RMSE of 1.1238. 3.8702, 1.9477 and 1.5720, 4.4419, 2.7657 for training, testing, and overall data sets. The ANN model performs well R^2^ of 0.9472, 0.9516 and 0.9493; MAE and RMSE of 1.7525, 2.6885, 2.0333 and 2.3382, 3.0877, 2.5859 for training, testing, and overall data sets. It is concluded that DT emerged as the most dependable model than SVM and ANN, while ANN shows lowest performance for predicting HC emissions^55^.

**Fig. 25:**
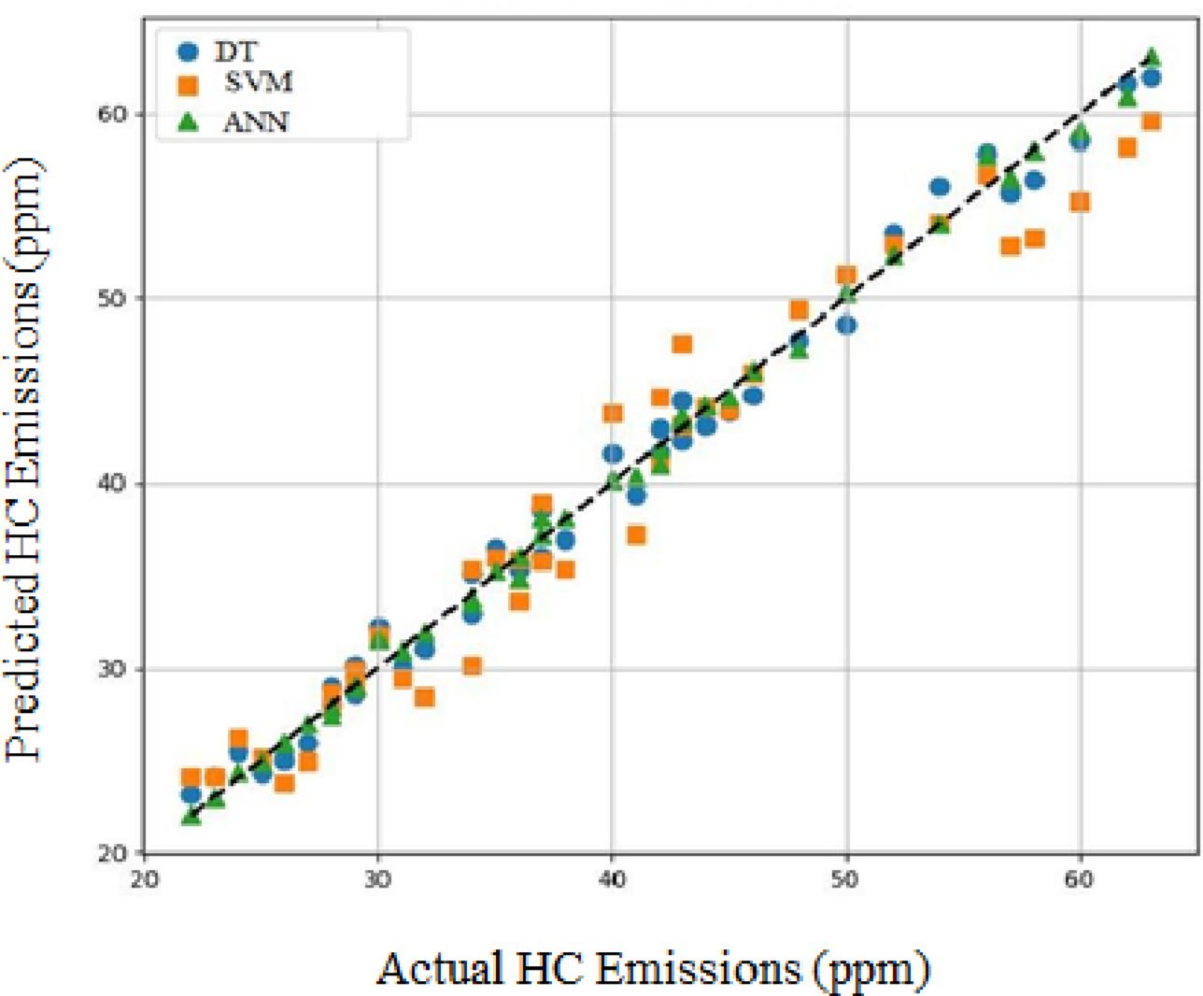
Predicted versus actual HC emissions of all models.

The predicted versus actual HC values for all models were shown in Fig. 25. The DT model exhibits a strong relation between predicted and actual HC emissions at all loads. At peak loads, the HMBD20 + MWCN100 fuel predicts HC emissions (43.33 ppm) identical with actual value (42 ppm) at 75% load. It shows ability of models while estimating the trends for nano additive blended fuels The SVM model predicts HC emissions with moderate accuracy especially at low to mid (25–50%) loads, but NP additive fuels exhibit consistent trends like experimental values. The ANN model represents a high consistency in predicting of HC emissions for all fuels. At 25% load, HMBD20 + ANP100 fuel shows the actual HC of 56 ppm while predicted value of 56.13 ppm confirms least error among remaining fuels^43^.

### NOx emissions

The performance of the DT, SVM and ANN (MLP) model to predict NOx emissions was evaluated using performance metrics. The Table 8 shows the Performance metrics of NO_x_ and Smoke emissions. The DT model was outperformed with highest R^2^ of 0.9981, 0.9685 and 0.9891, lowest MAE and RMSE of 10.39, 51.92, 22.85 and 14.65, 59.57, 34.85 for training, testing, and overall data sets. The SVM model shows good R^2^ of 0.9378, 0.8876 and 0.9226; MAE and RMSE of 64.60. 85.18, 70.78 and 83.11, 112.62,92.95 for training, testing, and overall data sets. The ANN model performs well R^2^ of 0.9997, 0.9571 and 0.9868; MAE and RMSE of 2.75, 59.52, 19.78 and 5.73, 69.59, 38.42 for training, testing, and overall data sets. Among all models, ANN was proved as the greater accuracy model compared to DT and SVM, while SVM exhibits moderate performance for predicting NOx emission^56^.

**Fig. 26:**
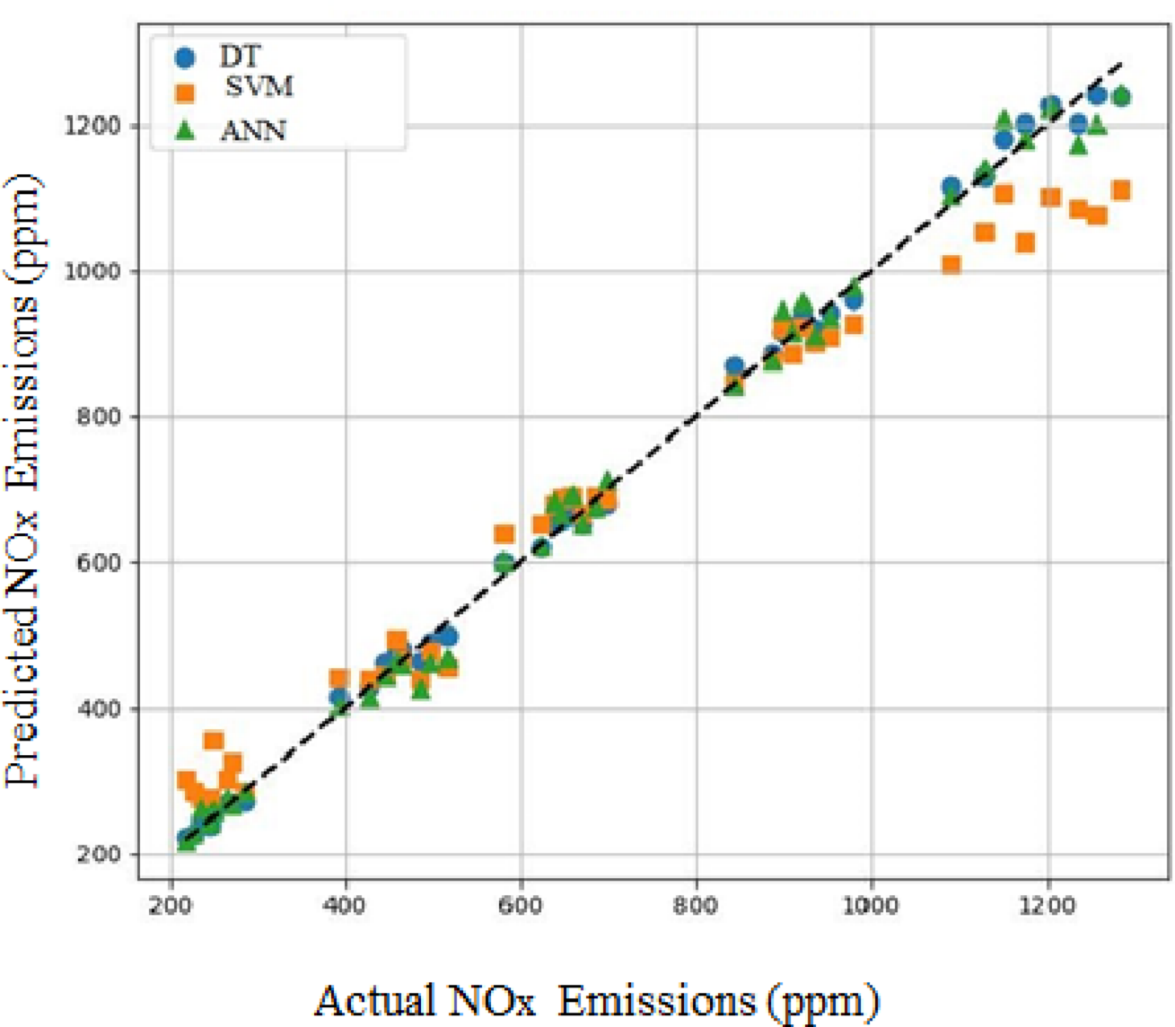
Predicted versus actual NOx emissions of all models.

Figure 26 depicts predicted versus actual NOx emissions of all models. This model exhibits a strong relation between predicted and actual NOx emissions at all loads. At peak loads, the HMBD20 + MWCN100 fuel predicts NOx emissions (982.5 ppm) very nearer to the actual value (980 ppm) which exhibits model predictive accuracy for nano additive blended fuels. The SVM model predicts NOx emissions with a reasonable accuracy, especially at high (75–100%) loads. The HMBD20 + TINP100 fuel at peak load shows a predicted value of 691 ppm whereas actual value is 686 ppm at 50% load confirms least error and the reliability of model^40^. The ANN model represents a high consistency in predicting NOx emissions for all fuels. At 50% load, HMBD20 + MWCN100 fuel shows the actual NOx of 698 ppm while predicted value of 697.67 ppm which confirms lowest deviation among all fuels^41^.

**Table 8:** Performance metrics of NOx, and smoke emissions.

Performance metrics		NOx			Smoke		
		DT	SVM	ANN	DT	SVM	ANN
R^2^	Training data	0.9981	0.9378	0.9997	0.9975	0.9851	0.9912
	Testing data	0.9685	0.8876	0.9571	0.9247	0.9099	0.9617
	Total data	0.9891	0.9226	0.9868	0.9808	0.9547	0.9844
MAE	Training data	10.39	64.60	2.75	0.7550	1.1344	1.60
	Testing data	51.92	85.18	59.52	4.3306	4.6453	3.02
	Total data	22.85	70.78	19.78	1.8277	2.1876	2.03
MSE	Training data	214.60	6907.10	32.80	1.1750	5.4395	4.20
	Testing data	3548.43	12682.83	4842.71	24.8543	52.4602	12.66
	Total data	1214.75	8639.82	1475.78	8.2788	19.5457	6.74
RMSE	Training data	14.65	83.11	5.73	1.0840	2.3323	2.05
	Testing data	59.57	112.62	69.59	4.9854	7.2429	3.56
	Total data	34.85	92.95	38.42	2.8773	4.4211	2.60

### Smoke emissions

The performance of the DT, SVM and ANN (MLP) model to predict smoke emissions were evaluated using performance metrics. The DT model was outperformed with highest R^2^ of 0.9975, 0.9247 and 0.9808, lowest MAE and RMSE of 0.7550, 4.3306, 1.8277 and 1.0840, 4.9854, 2.8773 for training, testing, and overall data sets. The SVM model shows good R^2^ of 0.9851, 0.9099 and 0.9547; MAE and RMSE of 1.1344, 4.6453, 2.1876 and 2.3323, 7.2429, 4.4211 for training, testing, and overall data sets. The ANN model performs well R^2^ of 0.9912, 0.9617 and 0.9844; MAE and RMSE of 1.60, 3.02, 2.03 and 2.05, 3.56, 2.60 for training, testing, and overall data sets. Among all models, DT emerged as the most dependable model followed by ANN, while SVM exhibits better performance for predicting smoke emissions^57^.

**Fig. 27:**
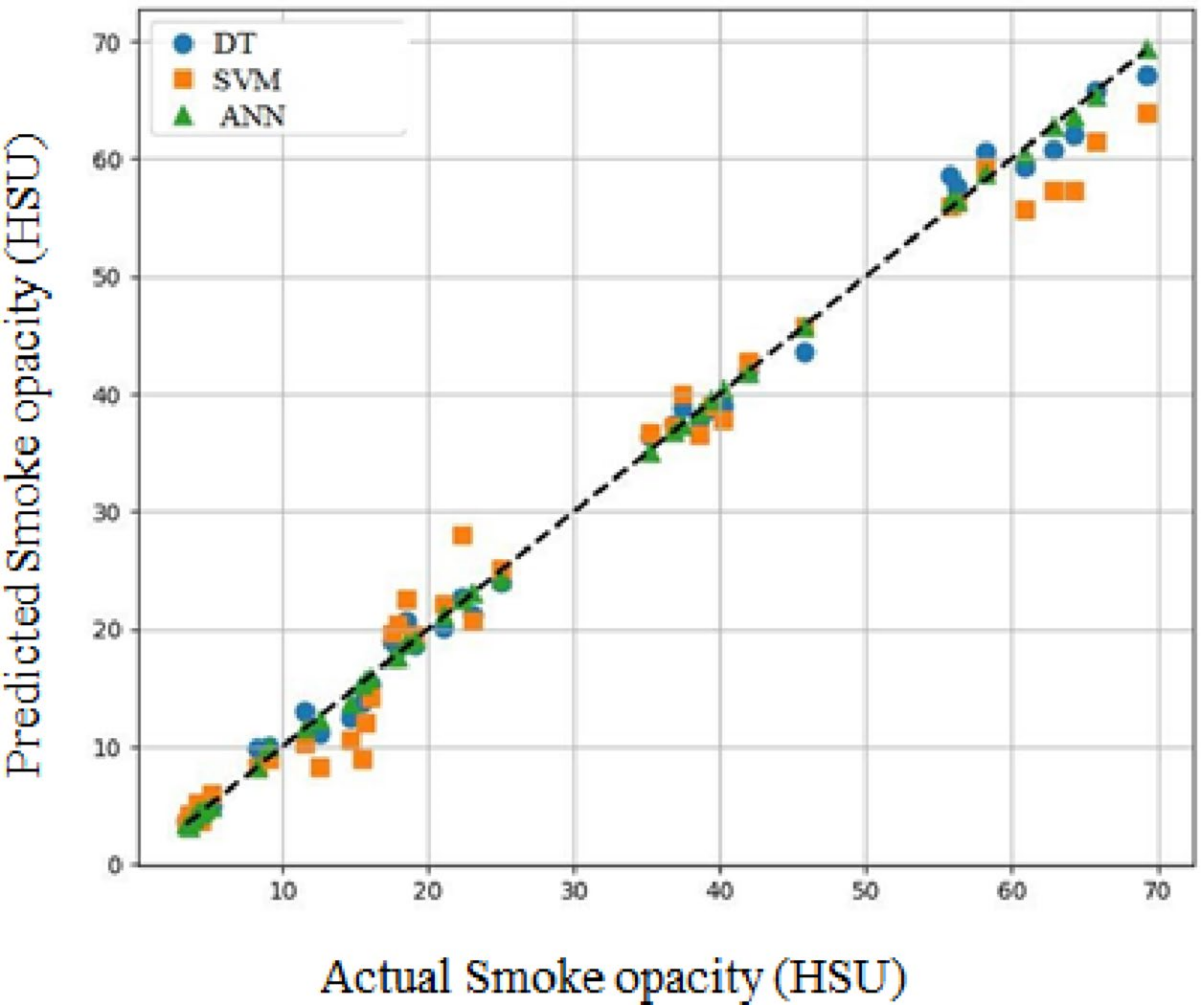
Predicted versus actual smoke emissions of all models.

Figure 27 was depicted for predicted versus actual smoke values of all models. The DT model exhibits a strong relation between predicted and actual smoke emissions at all loads. At peak load, the HMBD20 + TINP50 fuel predicts smoke emissions (62.63 HSU) nearer to the actual value (62.80 HSU) shows capability of model for predicting the output. The SVM model predicts smoke emissions with greater accuracy, especially at high (75–100%) loads and for nano particle blends^59^. The HMBD20 + TINP100 fuel at peak load shows a predicted value of 56.30 HSU and actual value is 56.20 HSU. The ANN model shows a moderate consistency in predicting smoke emissions for all fuels. At 75% load, HMBD20 + MWCN50 fuel shows the actual smoke value of 38.60 while a predicted value of 38.23 exhibits the lower deviation among all fuels^42^.

### Heat map analysis

**Fig. 28:**
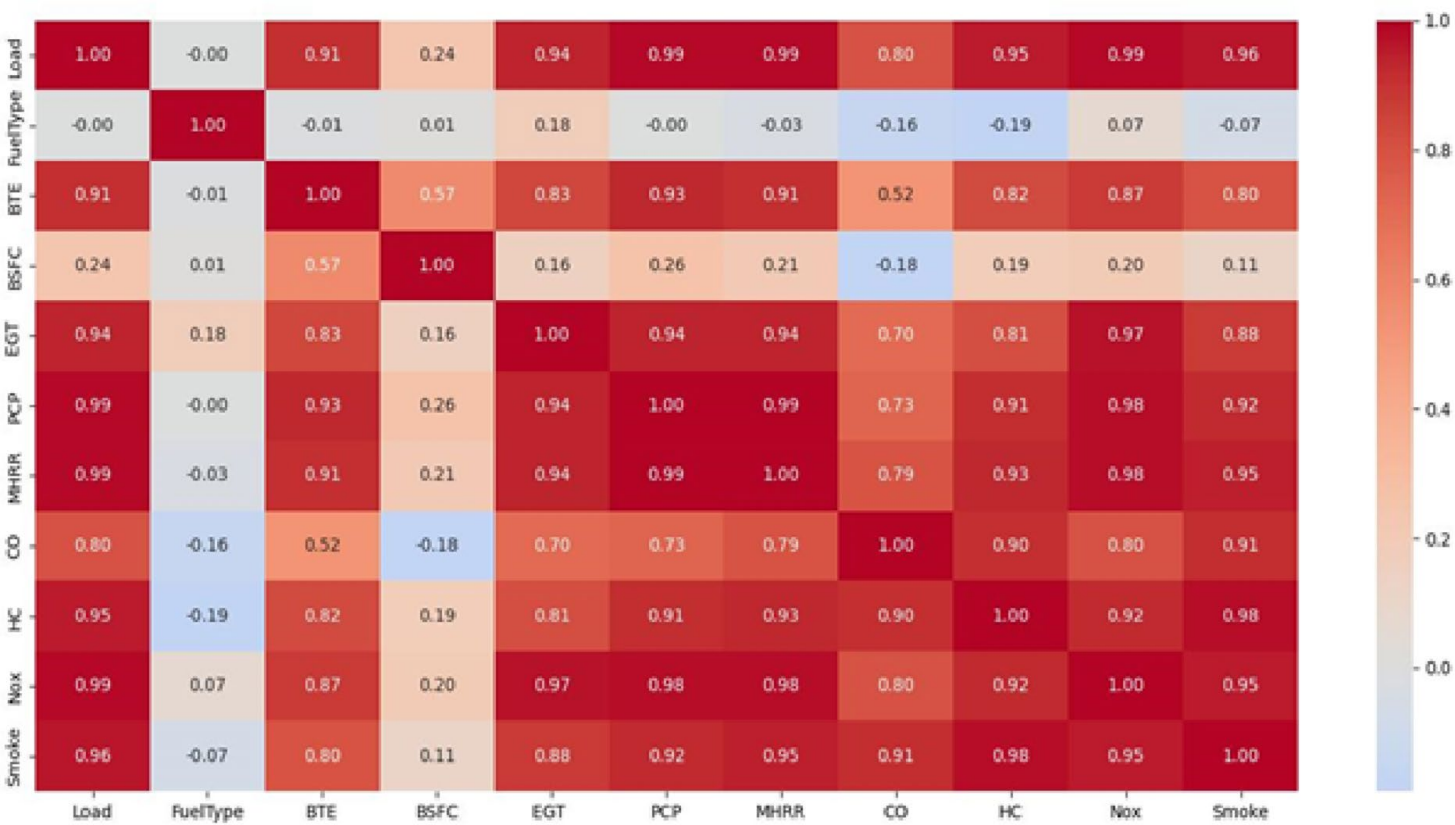
Correlation heat map for all engine parameters.

The Pearson correlation heatmap shown in Fig. 28 provides a statistical overview of the interrelations among engine parameters for different fuel types under varying load conditions. The intensity of color represents the degree of correlation with a strong positive value of + 1 and − 1 suggests that a strong inverse interaction. A strong positive correlation (> 0.9) was observed between load and engine parameters. It denotes that rise in engine load consistently increases the intensity of combustion. The engine load exhibits a strong positive correlation with BTE (0.91), PCP (0.94), MHRR (0.99), CO (0.99), NOx (0.95) and smoke (0.96) confirm the key role in the combustion and emission behavior. Next, fuel type shows lesser correlations such as BSFC (0.18) and EGT (0.21) indicates marginal influence along with negligible correlation with CO (−0.19) and HC (0.07) suggests that fuel composition plays minor role than load. A negative strong correlation exists between BTE and BSFC (−0.82) indicates that at higher efficiency results reduced fuel consumption along with a strong interdependence between CO and NOx emissions^53^.

### Pareto analysis

**Fig. 29:**
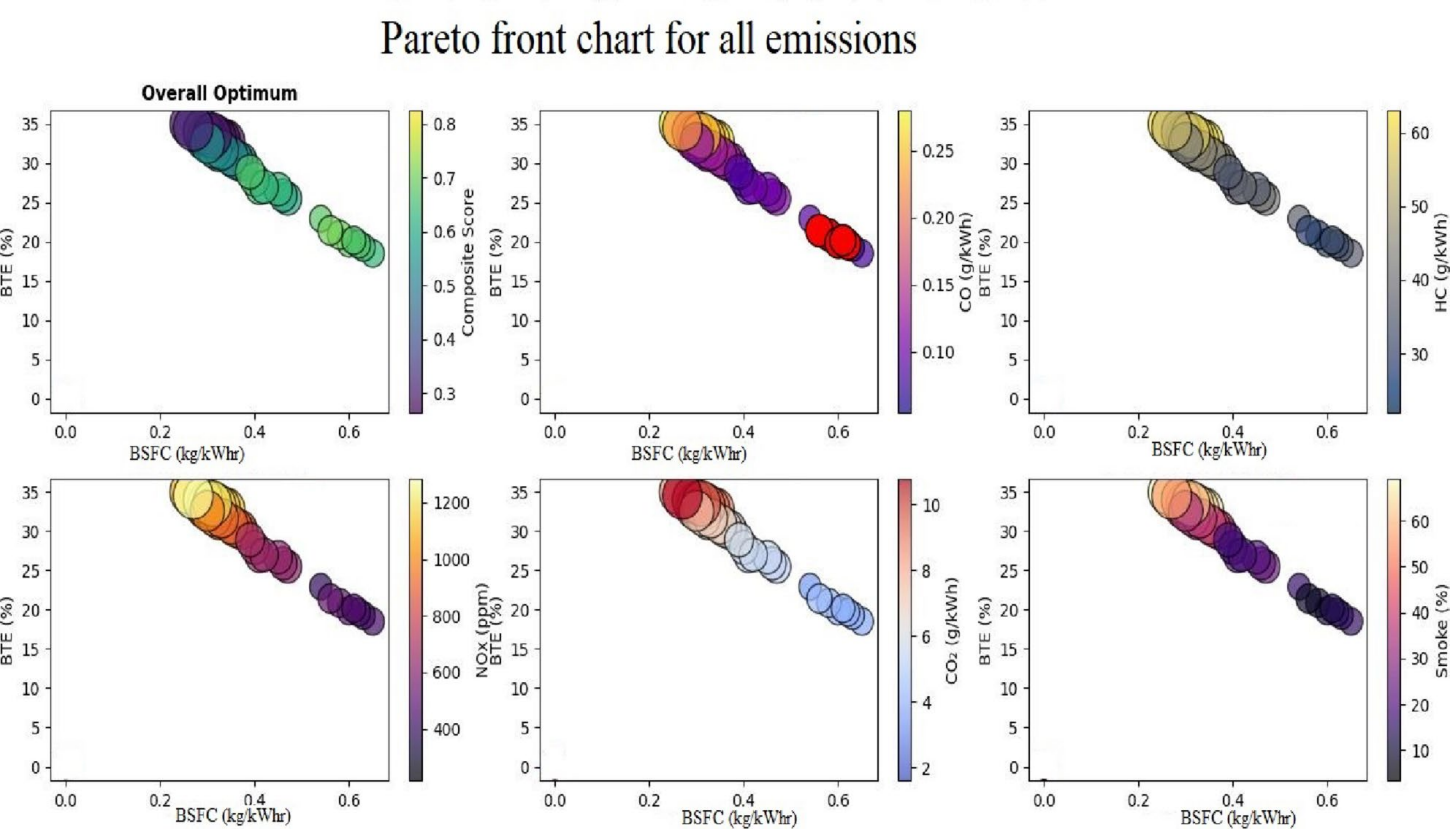
Pareto chart for engine parameters.

Figure 29 demonstrates the Pareto front analysis of the experimental data to find the optimal trade-offs between performance and emission parameters. In this Figure BTE is plotted against BSFC with the overall emission parameters along with subplots to represent the distribution of engine emissions under varying loads. The overall optimum subplot reveals all performance and emission parameters (CO, CO_2_, HC, NOx, smoke) into a single composite score to represent most balanced assessment between parameters. The multi-objective optimization approach is essential because of two objectives such as maximization of BTE and minimization of BSFC and emissions. The substantial reduction of CO and HC emissions in the subplots at lower and medium loads was reported due to enhanced oxidation and better atomization of NPs. The CO_2_ and smoke emissions reduction occurs at medium and high load conditions, but NOx emissions show an improvement with BTE because of higher temperatures observed for nano additive fuel blends^53^. Finally, the optimal condition was determined by the Pareto optimum method for HMBD20 + 100 ppm MWCNT fuel at 75% load which offers best results are BTE (32.56%), BSFC (0.30 kg/kWhr), CO (0.12%), HC (42 ppm), NOx (980 ppm), Smoke opacity (35.2 HSU) and CO_2_ (7.48%) among all fuel samples at different load conditions.

### Applicability domain analysis using Williams Plot

**Fig. 30:**
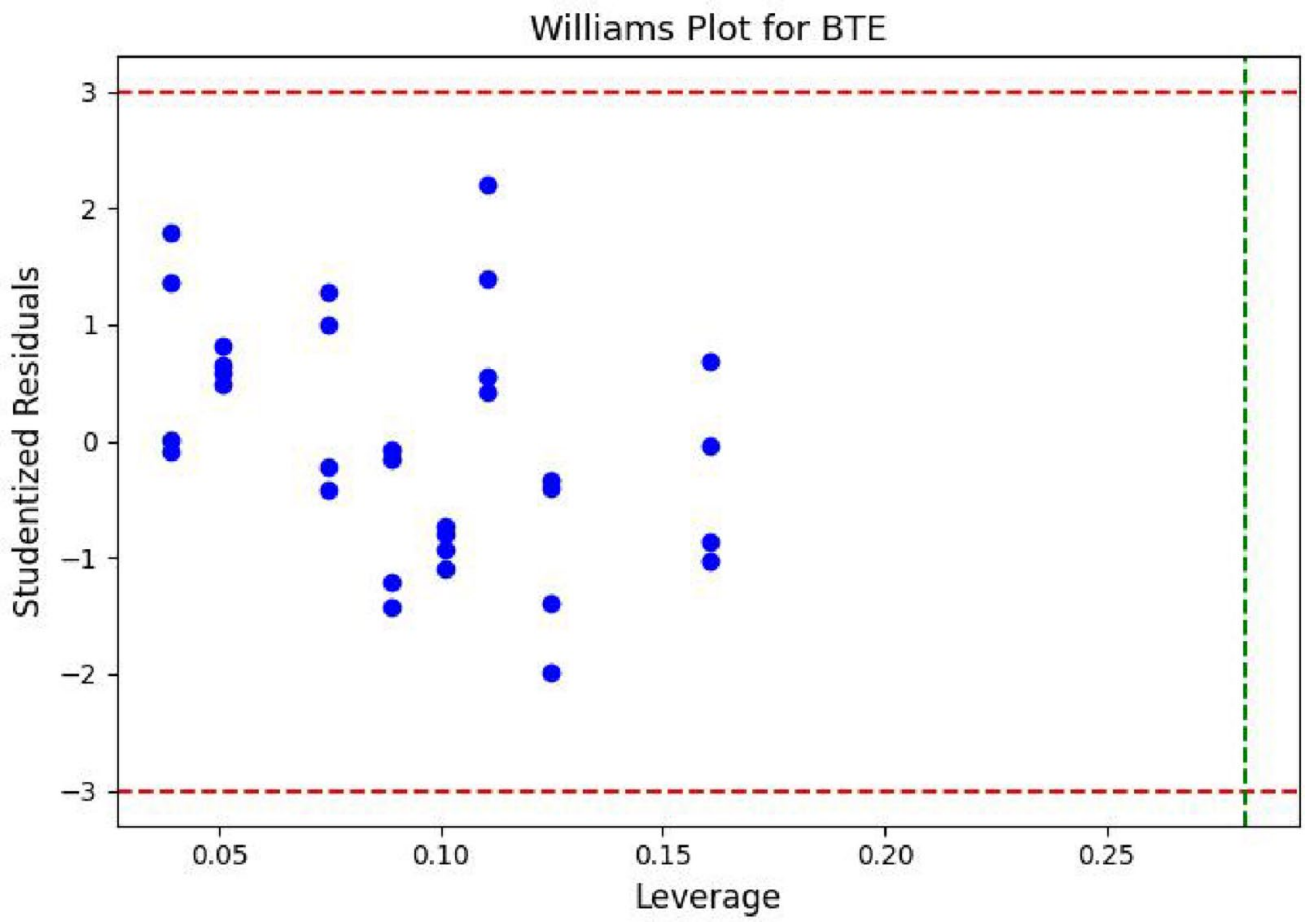
Williams plot for BTE.

The applicability domain and robustness of the developed models were analyzed by using the Williams plot, which represents the leverage values versus the studentized residuals. The warning leverage limit (h^*^) was calculated by using the following formula h^*^= (3(*p* + 1)/n). Where p is the number of input variables and n is the number of training data points. Data points with leverage values higher than the h indicates structurally influential observations, while those with studentized residuals greater than ± 3 are considered response outliers. It was observed that the majority of the data points fall within the acceptable region and confirm the reliability, stability, and good generalization capability of the developed models. The applicability domain of the developed model for BTE prediction was evaluated by using the Williams plot that was shown in Fig. 30. The warning leverage value (h^*^) was calculated as 0.281 based on the number of input variables and training samples. It can be observed that all data points lie within the acceptable region (|R| > 3 and h < h*) indicates that the absence of response outliers and structurally influential observations. It confirms that the developed model is robust, statically dependable and possesses a strong generalization capability for predicting BTE within the operating ranges^60^.

## Conclusions

The present study investigated the engine characteristics of a diesel engine fueled with hemp biodiesel blend (HMBD20) enriched with various nano additives at concentrations of 50 and 100 ppm. The AI based ML models were developed to predict engine responses based on the fuel type and engine load. The major observations of this study are as follows.

- The HMBD20 + MWCN100 blend achieved the highest BTE by 5.60% with a significant reduction in BSFC by 18.58% compared to HMBD20 fuel indicating an enhanced combustion efficiency and energy utilization.
- The nano additive fuels at 100 ppm exhibited an increased peak cylinder pressure and maximum heat release rate with HMBD20 + MWCN100 fuel showing the most pronounced improvement due to the intensified premixed combustion and superior heat transfer characteristics.
- The inclusion of TiO_2_ NP at 100 ppm to HMBD20 fuel results in a substantial reduction of CO (22.24%) and HC (16.12%) but an enhancement of NOx emissions by 13.7% due to elevated temperature inside the cylinder and improved oxidation.
- The DT model achieved superior accuracy for predicting BTE, BSFC, CO, HC, and smoke opacity with higher R^2^ value of 0.98 and lower RMSE.
- The ANN model results in the greater prediction accuracy for NOx emissions R^2^ of 0.9997 showing the capability of the model for non-linear combustion and emissions behavior.
- All the models exhibited acceptable minimal errors during the prediction of engine parameters using experimental datasets. It confirms the effectiveness of ML techniques and is suitable for predicting engine parameters such as performance and emissions.
- The optimal condition by the Pareto optimum method was recorded for the HMBD20 + 100 ppm MWCNT fuel at 75% load which offers best results are BTE (32.56%), BSFC (0.30 kg/kWhr), CO (0.12%), HC (42 ppm), NOx (980 ppm), Smoke opacity (35.2 HSU), and CO_2_ (7.48%).
- The warning leverage value (h^*^) was calculated as 0.281 and all data points lie within the acceptable limits of (|R| > 3 and h < h*) indicates the absence of response outliers and high leverage observations of the model for accurate BTE prediction within the operating range.

Finally, the integration of TiO_2_, Al_2_O_3_ and MWCNT nano additives with hemp biodiesel significantly enhances the diesel engine performance and particularly at high concentration of NP’s gives satisfactory results. The proposed machine learning models served as a powerful strategy for accurate and efficient prediction of engine parameters. The main limitations of the study are experiments that were conducted constant engine speed, evaluation of single blend ratio without considering long term stability of nano particles and developing machine learning models based on a small data set only. The following future studies were recommended for better analysis of diesel engines. These are the sedimentation and agglomeration of nano particles over time for fuel stability. Inclusion of additional parameters like fuel injection timing, fuel injection pressure, and different biodiesel blends for developing the better machine learning strategy. Adopting EGR or SCR with nano biodiesel blends for effective control of emissions especially for NOx and particular matter (PM) emissions.

## Data Availability

The datasets used and/or analyzed during the current study available from the corresponding author on reasonable request.

## Abbreviations

DI: Direct Injection
DT: Decision Tree
SVM: Support Vector Machine
ANN: Artificial Neural Network
R^2^: Co-efficient of Determination
MAE: Mean Absolute Error
MSE: Mean Squared Error
RMSE: Root Mean Squared Error
PCP: Peak cylinder pressure
MHRR: Maximum Heat release rate
ppm: parts per million
CO: Carbon Monoxide
HC: Hydrocarbons
NOx: Oxides of Nitrogen
HMBD20: 20% of Hemp Biodiesel + 80% of diesel
ANP: Al_2_O_3_ nano particle
TINP: TiO_2_ nano particle
MWCN: Multiwalled carbon nano tubes
EGT: Exhaust Gas Temperature
HSU: Hartridge Smoke Unit
FTIR: Fourier Transform Infrared Spectroscopy
SEM: Scanning Electron Microscopy
h^*^: Warning leverage value
R: Studentized residual
